# Sufficient component cause simulations: an underutilized epidemiologic teaching tool

**DOI:** 10.3389/fepid.2023.1282809

**Published:** 2023-11-10

**Authors:** Katrina L. Kezios, Eleanor Hayes-Larson

**Affiliations:** ^1^ Department of Epidemiology, Mailman School of Public Health, Columbia University, New York, NY, United States; ^2^ Department of Epidemiology, Fielding School of Public Health, University of California, Los Angeles, CA, United States

**Keywords:** simulation, causal inference, bias, sufficient component cause, teaching, confounding, selection bias, effect measure modification

## Abstract

Simulation studies are a powerful and important tool in epidemiologic teaching, especially for understanding causal inference. Simulations using the sufficient component cause framework can provide students key insights about causal mechanisms and sources of bias, but are not commonly used. To make them more accessible, we aim to provide an introduction and tutorial on developing and using these simulations, including an overview of translation from directed acyclic graphs and potential outcomes to sufficient component causal models, and a summary of the simulation approach. Using the applied question of the impact of educational attainment on dementia, we offer simple simulation examples and accompanying code to illustrate sufficient component cause-based simulations for four common causal structures (causation, confounding, selection bias, and effect modification) often introduced early in epidemiologic training. We show how sufficient component cause-based simulations illuminate both the causal processes and the mechanisms through which bias occurs, which can help enhance student understanding of these causal structures and the distinctions between them. We conclude with a discussion of considerations for using sufficient component cause-based simulations as a teaching tool.

## Introduction

Simulation is an important tool in epidemiologic teaching, especially for helping students understand causal inference (1). Although it is often used as a heuristic for causation, the sufficient component cause (SCC) framework has only rarely been used for simulation work [e.g., (2, 3)]. However, simulations based in the SCC framework have substantial potential for helping students understand causal mechanisms and sources of bias. In this article, we aim to make SCC-based simulations more accessible to epidemiology students and educators.

We assume some prior introduction to the SCC and potential outcomes (counterfactual) frameworks for causation, but briefly summarize these frameworks and the connection between them [for more detailed expositions see: (4–11)]. Next, we provide an overview of SCC-based simulations, including simple examples with sample R code, illustrating their utility for understanding and distinguishing between causation, confounding, selection bias, and effect modification. We conclude with discussion of considerations regarding sufficient component cause-based simulations as a teaching tool.

### Summary of SCC framework

In the SCC framework (4, 12, 13), a “cause of a specific disease occurrence is an antecedent event, condition, or characteristic that was necessary for the occurrence of the disease at the moment it occurred, given that other conditions are fixed.” (4). The assumptions of this framework include that no single cause is sufficient to yield the outcome on its own; rather, every cause works with and indeed requires other causes (“causal complements”), in causing the outcome. A set of causes (“components”) that are sufficient to cause the outcome are non-redundant (i.e., all components are needed). In addition, there are many possible sets of causal components that are sufficient to cause the outcome, making any specific set unnecessary (4). The first set of causal components to be completed is responsible for the outcome occurring, but individuals may complete multiple (redundant) sufficient sets by the time they are observed; this leads to discrepancy between the etiologic and excess fractions [see (2, 14, 15) for more details].

**Figure 1:**
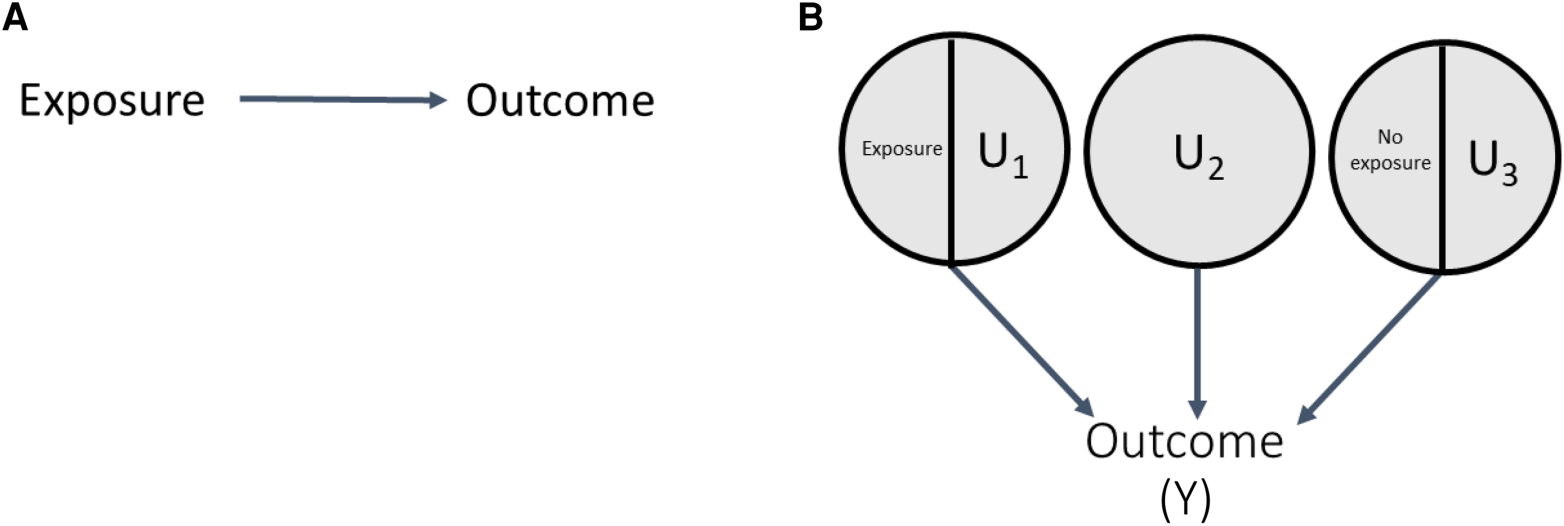
DAG and SCC model correspondence.

To illustrate this, consider the directed acyclic graph (DAG) in Figure 1A showing an exposure causing an outcome. The corresponding SCC model is shown in Figure 1B; each circle represents a sufficient set of causes (hereafter, a sufficient cause). The circle containing exposure and U1 indicates that the exposure alone is insufficient to cause the outcome (it requires the presence of other causal complements, some likely unknown, the totality of which are represented by “U1”). The circle containing U2 indicates that the outcome can additionally arise from causes unrelated to the exposure, all represented by “U2”. Finally, the exposure could also prevent the outcome, indicated by the sufficient cause including the absence of the exposure (i.e., no exposure) and its causal complements “U3”.

### Connection between the SCC framework and potential outcomes

The SCC and potential outcomes (PO) frameworks can be connected via response types. Let “E” and “Y” represent an exposure and outcome, respectively. Response types are labels defined for an individual by their set of potential outcomes under two or more exposure conditions (e.g., *Y^e^*^ = 1^ and *Y^e^*^ = 0^ are an individual's set of potential outcomes under exposure values of 1 and 0). Response types are specific to a set of exposures, outcome, and timeframe (i.e., observation period) (16). As shown in Table 1, for a dichotomous exposure, individuals who will experience the outcome in the specified timeframe regardless of exposure (i.e., *Y^e^*^ = 1 ^= 1 and *Y^e^*^ = 0 ^= 1) are said to be “doomed”, while individuals who experience the outcome only if exposed (i.e., *Y^e^*^ = 1 ^= 1 and *Y^e^*^ = 0 ^= 0) are said to be “causal.” “Preventive” response types are individuals who experience the outcome only if unexposed (exposure prevents the outcome, i.e., *Y^e^*^ = 1 ^= 0 and *Y^e^*^ = 0 ^= 1), and individuals who never experience the outcome, regardless of exposure. (i.e., *Y^e^*^ = 1 ^= 0 and *Y^e^*^ = 0 ^= 0), are said to be “immune.”

**Table 1:** Correspondence between potential outcomes and SCC models for example in Figure 1.

Individual's response type	*Y^e^* ^ = 1^	*Y^e^* ^ = 0^	Individual's characteristics (risk set type)^a^
Doomed	1	1	U1_(1)_U2_(1)_U3_(1)_ U1_(1)_U2_(1)_U3_(0)_ U1_(0)_U2_(1)_U3_(1)_ U1_(0)_U2_(1)_U3_(0)_ U1_(1)_U2_(0)_U3_(1)_
Causal	1	0	U1_(1)_U2_(0)_U3_(0)_
Preventive	0	1	U1_(0)_U2_(0)_U3_(1)_
Immune	0	0	U1_(0)_U2_(0)_U3_(0)_

*Y^e^*^ = 1^, potential outcome under exposure = 1; *Y^e^*^ = 0^, potential outcome under exposure = 0.

^a^ Risk set notation: U1_(1)_ indicates the individual has causal component U1, while U1_(0)_ indicates they do not have U1.

An individual's response type is determined by their distribution of component causes *other than* the exposure of interest [i.e., their “risk set type” (14)]. Table 1 shows the correspondence between response types and risk set types for the example in Figure 1. Any individuals with U2 (or both U1 and U3), are “doomed” response types because they complete a sufficient cause of the outcome regardless of their exposure status. Individuals with the causal complement of exposure, U1, who are not doomed (i.e., lack components to complete any other sufficient causes; i.e., U2 and U3) are “causal” types. Conversely, individuals with the causal complement of absence of exposure, i.e., U3, who are not doomed are “preventive” types. Finally, individuals who possess neither U1 nor U2 nor U3, and therefore cannot complete any sufficient causes of the outcome regardless of exposure status, are “immune” types. Theory that the exposure can only affect the outcome in one direction is called monotonicity; in these cases, either causal or more commonly, preventive types, are assumed to not exist. Such monotonicity may occur due to the lack of a preventive sufficient cause mechanism (e.g., the No exposure/U3 sufficient cause in Figure 1 would not exist), or a weaker assumption that there are no individuals in the sample who would complete only the sufficient cause including No exposure/U3 (11, 17).

The distribution of response types in the population determines the size of the effect of an exposure or intervention in that population. Because response types are determined by the risk set type, the SCC framework makes clear that the true size (i.e., magnitude) of a causal effect is driven by the prevalence of the component causes other than the exposure of interest in the population (4).

In addition to clarifying magnitude of causal effects, response types also help clarify when to expect bias in an unadjusted effect estimate. We define bias as a difference in an estimated causal effect and the true causal effect that arises due to non-exchangeability (different distribution) of response types between exposed and unexposed individuals.

## Methods

### Overview of SCC-based simulations

**Figure 2:**
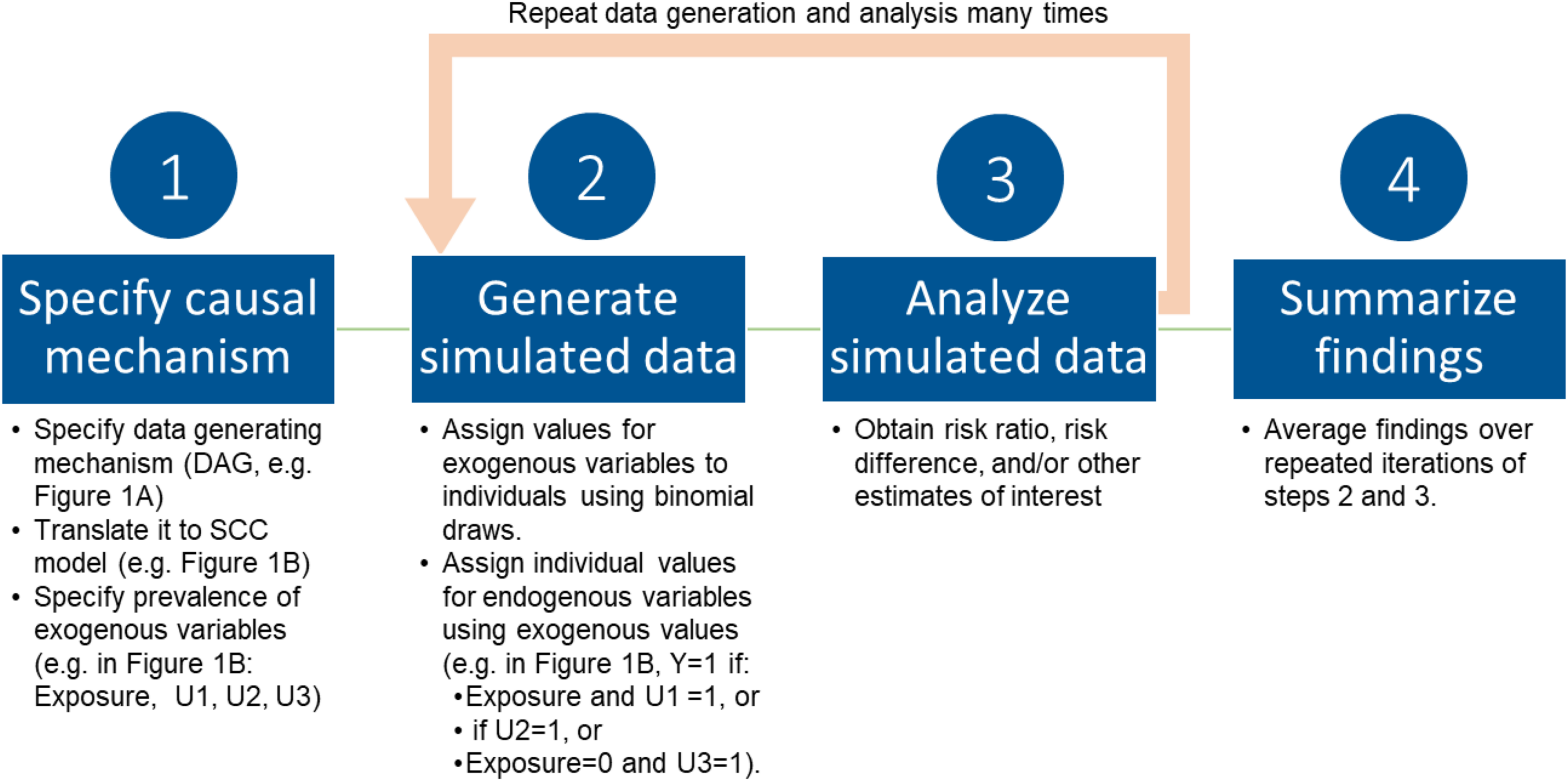
Overview of SCC simulation approach.

Simulations based in the SCC framework were developed to understand the causal structures and mechanisms in which bias occurs (15, 18), by generating a sample for which all causal components, and therefore distribution of response types and magnitude of true causal effects, are known. In this section and Figure 2, we provide an overview of the simulation steps; illustrative examples are provided in a later section. In this approach, we begin with a DAG, typically of dichotomous variables, and translate it to an SCC model by including additional variables, such as causal complements and other sufficient causes as needed to meet the SCC framework assumptions outlined above (Figure 2, step 1) (5, 15, 18). For example, for the DAG in Figure 1, translation to the SCC model involves: (i) a sufficient set including the Exposure, with the additional variable U1 representing causal complements of Exposure required for SCC conditions; (ii) a sufficient set with No exposure and the additional variable U3 representing causal complements of No exposure; and (iii) an additional variable U2, representing other sufficient causes of the outcome not related to exposure. Next, the prevalences of all exogenous variables (variables without a parent) in the SCC model are specified, which determines the true magnitude of the causal effect of interest as discussed above. These prevalences also define the effect sizes for other relationships between variables in the DAG (e.g., strength of effect of a confounder on the exposure and outcome). Importantly, in contrast to regression-based simulations where effect sizes are input directly as coefficients in data-generating models, in SCC simulations the prevalences of exogenous causal components are set to produce the desired prevalence of the outcome and effect sizes (additional details and example calculation in Supplementary Appendix 1).

Next (Figure 2, step 2), individual-level data are generated, with values for variables assigned in a hierarchical fashion, starting with exogenous variables and working toward the outcome, the endogenous variable with the most parents in the figure. Individual values for exogenous variables are drawn from binomial probability distributions for the specified sample prevalences. For each individual, values for endogenous variables (e.g., outcome and any other variables with a parent in the original DAG) emerge from the values of the components that cause them. This results in a dataset in which the values of all causal components (variables) for each individual are known. This dataset can then be analyzed in accordance with the study question as one would analyze any real-world dataset (Figure 2 step 3). The data generation process is then repeated many times, with results summarized across iterations (Figure 2, step 4). Observed results can be compared to the known true magnitude of the causal effect.

### Illustrative examples

We illustrate SCC-based simulations showing causation, confounding, selection (collider) bias, and effect modification using the example that low educational attainment (e.g., less than high school degree) is positively associated with dementia risk (19–21). For each causal structure, we include translation from the DAG to SCC model, comparison of true and observed results, and discussion of response types to illuminate bias mechanisms. To illustrate how different causal structures can lead to similar observed associations in data, in all simulations we set values for causal components to produce crude risk ratios of approximately 1.55, which falls in the range of associations reported in the empirical literature on education and dementia (19–21). A key benefit of simulations, especially in the context of teaching epidemiologic concepts, is that they allow the analyst to specify the truth, and do not need to perfectly replicate the real world; the causal structures we show are intentionally simplified, allowing us to isolate each one and demonstrate how it gives rise to associations and patterns in data. We note that simulations may be used for other purposes, such as answering applied research questions, where specification of accurate DAGs and effect sizes is more important, but this is beyond the scope of this paper.

For all scenarios, we simulate 1,000 samples of 10,000 individuals, and in each scenario, our estimand is the average treatment effect (ATE) in the analytic sample. All code is available in the Supplementary Material (Supplementary Appendix 5) and on GitHub (https://github.com/ehayeslarson/SCCsims), and is intended to accompany the text.

For these examples, we make several simplifying assumptions. First, unless noted otherwise by the DAG, causal components (e.g., causal complements of the exposure) are independent from one another. In all example simulations, we invoke monotonicity, positing that low education cannot prevent dementia in our sample (i.e., here we assume the No Exposure/U3 cause in Figure 1B does not exist, although one could instead invoke the weaker monotonicity assumption described above without changing results). These assumptions can be relaxed, but add complexity to the simulations that is beyond the scope of this paper.

## Results

### Causation

**Figure 3:**
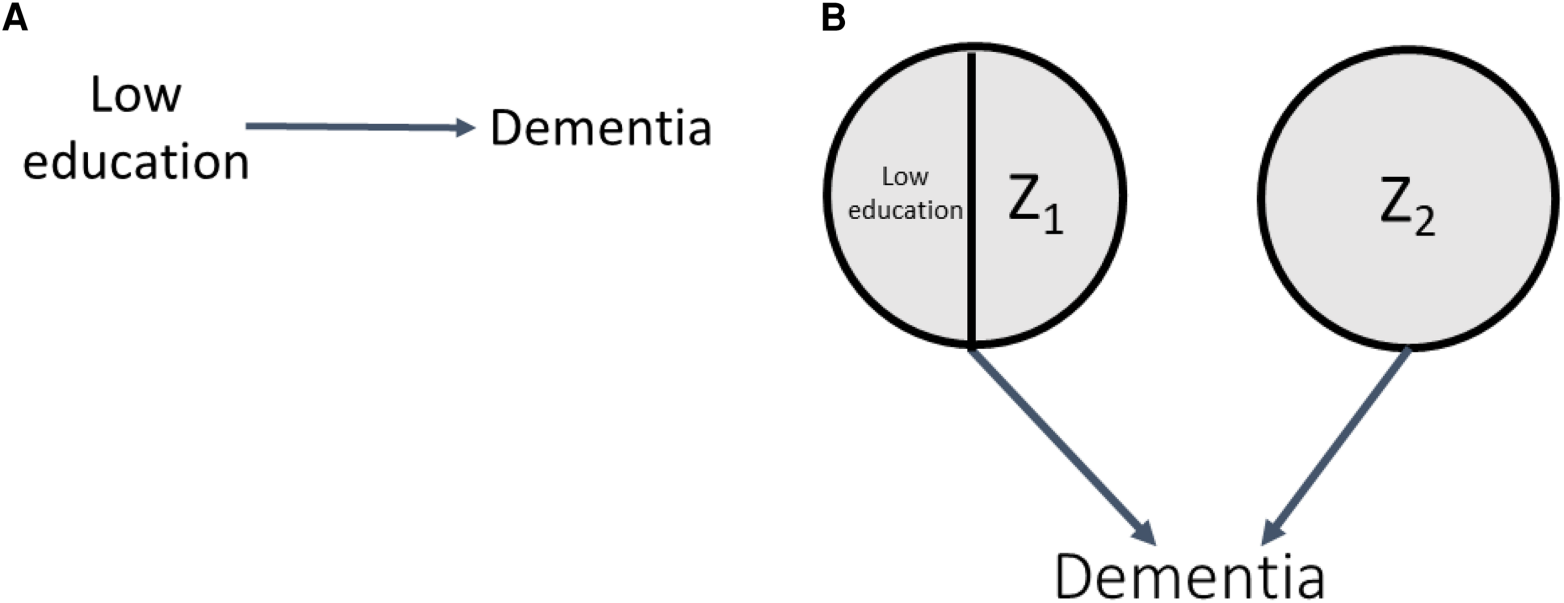
DAG and SCC model for causation.

As discussed above, one possible reason for the association between low education and dementia is that low education is a cause of dementia (DAG and SCC model shown in Figures 3A,B, respectively) (19). For this causal model, response types corresponding to causal components are shown in Table 2.

**Table 2:** Distribution of response types in simulations demonstrating causation.

Individual's response type	Individual's characteristics (risk set type)^a^	Prevalence of RT in those with low education (*E*+)	Prevalence of RT in those with high education (*E*-)
Doomed	Z1_(1)_Z2_(1)_ Z1_(0)_Z2_(1)_	10.0% (9.3%–10.6%)	10.0% (9.0%–11.1%)
Causal	Z1_(1)_Z2_(0)_	5.4% (4.9%–6.0%)	5.4% (4.6%–6.2%)
Preventive	N/A	N/A	N/A
Immune	Z1_(0)_Z2_(0)_	84.6% (83.8%–85.4%)	84.5% (83.2%–85.8%)

RT, response type; *E*+, exposed; *E*-, unexposed; N/A, not applicable.

^a^ Risk set notation: Z1_(1)_ indicates the individual has causal component Z1, while Z1_(0)_ indicates they do not have Z1.

In Section 1 of the accompanying simulation code, we simulate this scenario, setting the prevalences of Z1 and Z2 to values that produce a true risk ratio (RR) of 1.54 and a true risk difference (RD) of 0.054 (5.4%) (0.06 and 0.10, respectively; code Section 1A). As a reminder, in SCC simulations, true effect sizes for all relationships in the DAG result from probabilities corresponding to the specified distributions of the exogenous causal components, and the components can be set to result in an *a priori* effect size of interest (here, for example, RR of 1.54). Under monotonicity, the causal effect is determined by the prevalence of the causal complements of the exposure and the prevalence of doomed response types. For example, in this simulation, where we specify no bias (here, full exchangeability is met) and *Z*1 and *Z*2 are independent, the true ATE of low education on dementia is given by:

$$\begin{array}{r} \mathrm{True}\;\mathrm{risk}\;\mathrm{ratio}\;(\mathrm{RR})=\;\frac{P(Y^{\mathrm{low}\;\mathrm{ed}=1})}{P(Y^{\mathrm{low}\;\mathrm{ed}=0})}=\;\frac{P(Z1)+P(Z2)-P(Z1\;\&\;Z2)}{P(Z2)} \end{array}$$

$$\begin{array}{rl} \mathrm{True}\;\mathrm{risk}\;\mathrm{difference}\;(\mathrm{RD}) & =\;P(Y^{\mathrm{low}\;\mathrm{ed}=1})\;-\;P(Y^{\mathrm{low}\;\mathrm{ed}=0})\\ & =(P(Z1)+P(Z2)-P(Z1\;\&\;Z2))-P(Z2) \end{array}$$

Inserting prevalences of 0.06 and 0.10 for components *Z*1 and *Z*2, respectively, into these equations produces a true RR of 1.54 and a true RD of 0.054, as desired. A value of 0.10 was chosen for *Z*2 (which represents the marginal prevalence of the outcome in the unexposed), because 10% is a plausible level of dementia in older adults without low education. Prevalence of *Z*1 was back-calculated from the prevalence of *Z*2 and the desired risk ratio of 1.55. Supplementary Appendix 1 includes a detailed derivation of the RR and RD and shows how back calculations can be performed to obtain alternative specifications of *Z*1 or *Z*2 given a different desired effect size.

Across simulated samples (code Section 1B), as expected, the observed effect estimates in the sample closely matched the specified truth (Section 1C): the observed mean RR (with 95% confidence interval [CI]) was RR_crude_ = 1.54 (1.36–1.73), and the RD_crude_ was 0.054 (95% CI 0.039–0.067). Section 1D of the accompanying code looks “under the hood” of the simulation to obtain the prevalence of individuals of different response types in the sample. The average distribution of these response types across the 1,000 simulated samples stratified by exposure is shown in Table 2. The mean prevalence of “causal” types is 5.4%, matching the RD, as the proportion of causal types represent the proportion of the sample in whom the exposure has an effect under our assumption of monotonocity. Importantly, the simulation shows that the distribution of response types is balanced between those simulated to have low and high education, implying exchangeability (no bias). We note that in this example, full exchangeability is met (doomed, causal, and immune all comparable between exposed and unexposed), but partial exchangeability is sufficient for some measures (22).

### Confounding

**Figure 4:**
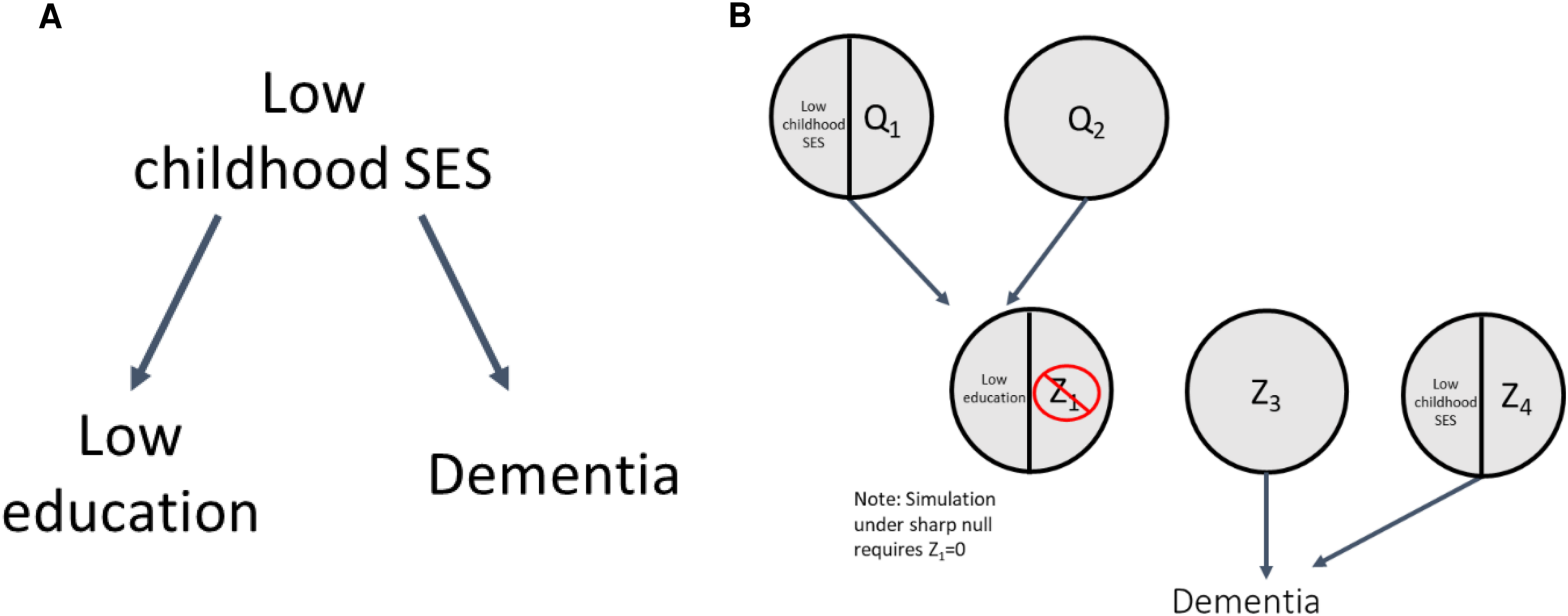
DAG and SCC model for confounding.

Another explanation for the low education and dementia association could be confounding by low childhood socioeconomic status (SES) (23). This is shown by the DAG in Figure 4A; low education is no longer exogenous, as it is caused by low childhood SES, and there is no true effect of low education on dementia. As a result, the corresponding SCC model, shown in Figure 4B, has an additional level of sufficient causes. Both low childhood SES/*Q*1 and *Q*2 are sufficient causes for low education, representing the arrow in the DAG from low childhood SES to low education. The circles with *Z*3 and low childhood SES/*Z*4 make up the sufficient causes for dementia not including low education; the latter represents the arrow in the DAG from low childhood SES to dementia. To illustrate how confounding can create an association between exposure and outcome even if, in truth, no causal relationship exists between them, we perform simulations under the sharp null (illustrated by the absence of an arrow between low education and dementia in Figure 4A).

Section 2 of the accompanying code shows simulations illustrating confounding. To simulate the sharp null as noted above, we set the prevalence of *Z*1 to be 0, such that no individuals could complete the sufficient cause for dementia involving low education and *Z*1; this results in a true RR = 1 and true RD = 0 (no true effect of low education on dementia). We then specified prevalences of other causal components (code Section 2A) to generate confounding and the desired crude RR of 1.55 (within each simulated sample, individuals' exposure and outcome values were determined by their values of the exogenous components, see code Section 2B). Correspondingly, the crude RR and RD observed across the simulated samples were 1.55 (1.42–1.71) and 0.079 (0.064–0.094), respectively (code Section 2C).

The mechanism by which confounding causes bias is clearly shown by the distribution of the response types between the exposed and unexposed in Table 3 (code Section 2D). Recall that because we set *Z*1 = 0 there are *no* causal response types in our sample (as expected from the simulation under the sharp null). Thus, any cases of dementia in the sample are due to doomed response types, and any association between the exposure and dementia is due to imbalance of doomed types across the exposed and unexposed. Table 3 shows there are more “doomed” response types among the exposed than unexposed (22.2% vs. 14.3%), increasing the prevalence of the outcome in that group, resulting in a crude association between low education and dementia that is greater than 1. The imbalance in the doomed response types occurs because low childhood SES causes both low education and dementia; as a result, among those with low education, more individuals have low childhood SES and complete the doomed sufficient cause involving low childhood SES and *Z*4. Our crude estimates reflect this bias; to obtain the causal effect of low education on dementia we have to adjust for this confounding, which can be done via standardization (among other methods). After standardizing to the distribution of the confounder, low SES (code Section 2C), the adjusted RR and RD match the true null effects [RR_adj _= 1.00 (0.90–1.11)] and RD_adj _= 0.000 [−0.022–0.019]). As a companion to simulation results, Supplementary Appendix 2 includes detailed mathematical derivation of the crude and adjusted RR and RD values. In addition, an illustrative simulation of confounding not under the sharp null (i.e., when there is a non-zero exposure effect) is available in Supplementary Appendix 2 and the accompanying code.

**Table 3:** Distribution of response types in simulations demonstrating confounding.

Individual's response type (effect of low education on dementia)	Individual's characteristics (risk set type)	Prevalence of RT in those with low education (*E*+)	Prevalence of RT in those with high education (*E*-)
Doomed	Z1_(1)_Z3_(1)_Z4_(1)_LowSES_(1)_ Z1_(0)_Z3_(1)_Z4_(1)_LowSES_(1)_ Z1_(1)_Z3_(1)_Z4_(1)_LowSES_(0)_ Z1_(0)_Z3_(1)_Z4_(1)_LowSES_(0)_ Z1_(1)_Z3_(1)_Z4_(0)_LowSES_(1)_ Z1_(0)_Z3_(1)_Z4_(0)_LowSES_(1)_ Z1_(1)_Z3_(1)_Z4_(0)_LowSES_(0)_ Z1_(0)_Z3_(1)_Z4_(0)_LowSES_(0)_ Z1_(1)_Z3_(0)_Z4_(1)_LowSES_(1)_ Z1_(0)_Z3_(0)_Z4_(1)_LowSES_(1)_	22.2% (21.2%–23.2%)	14.3% (13.3%–15.5%)
Causal^b^	Z1_(1)_Z3_(0)_Z4_(0)_LowSES_(0)_ Z1_(1)_Z3_(0)_Z4_(1)_LowSES_(0)_ Z1_(1)_Z3_(0)_Z4_(0)_LowSES_(1)_	0%	0%
Preventive	N/A	N/A	N/A
Immune	Z1_(0)_Z3_(0)_Z4_(0)_LowSES_(0)_ Z1_(0)_Z3_(0)_Z4_(1)_LowSES_(0)_ Z1_(0)_Z3_(0)_Z4_(0)_LowSES_(1)_	77.8% (76.8%–78.8%)	85.7% (84.5%–86.7%)

RT, response type; *E*+, exposed; *E*-, unexposed; N/A, not applicable.

^a^ Risk set notation: Z1_(1)_ indicates the individual has causal component Z1, while Z1_(0)_ indicates they do not have Z1.

^b^ In the simulation, Z1 was set to zero to simulate under the sharp null.

### Selection (collider) bias

**Figure 5:**
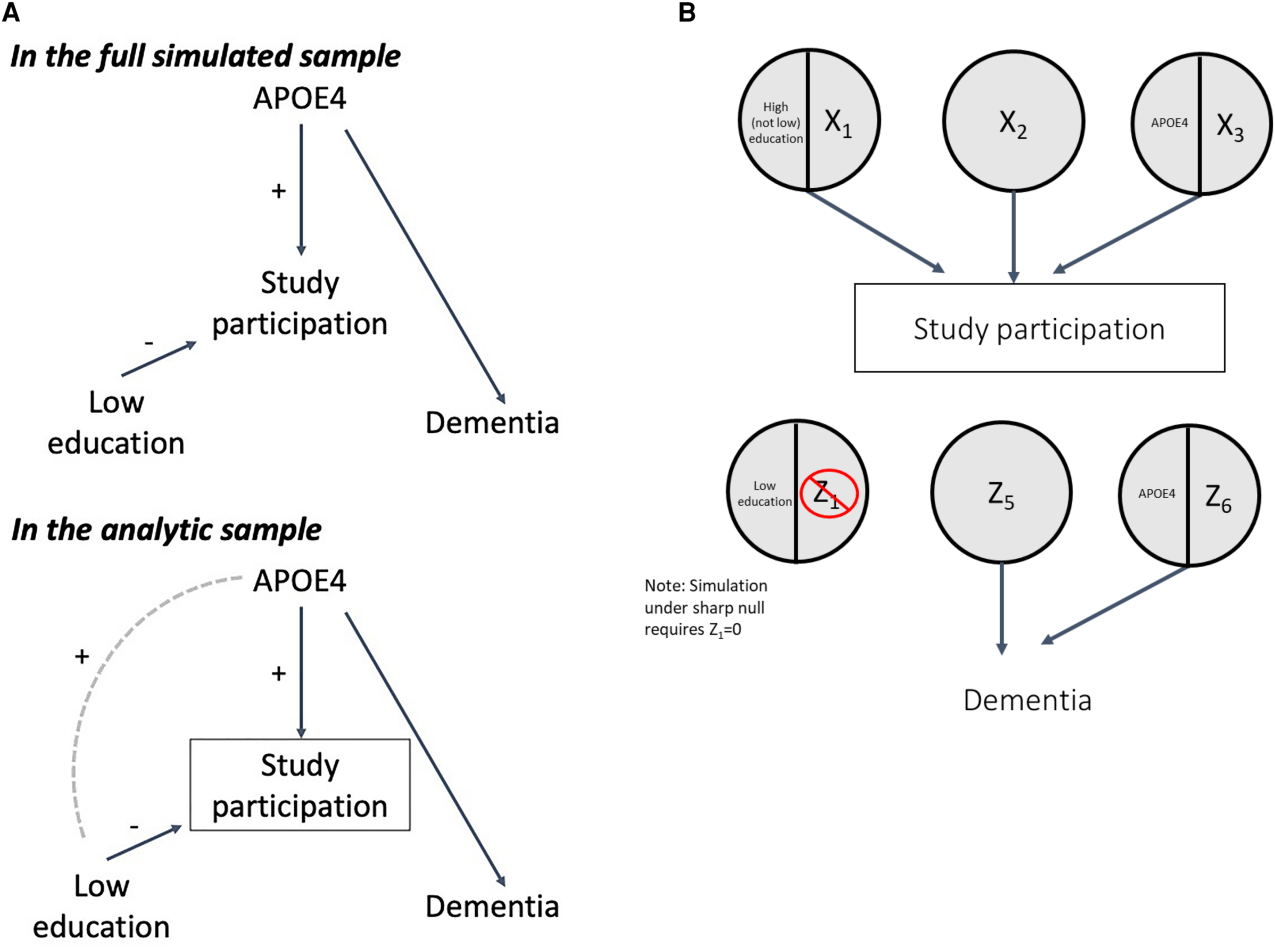
DAG and SCC model for collider bias.

Another reason that low education and dementia could be associated is selection bias (i.e., collider bias), as individuals with certain characteristics including high education and family history of dementia are often overrepresented in dementia cohorts (24–27). We will illustrate this in the DAG shown in Figure 5A: low education prevents (i.e., high education causes), and the APOE ε4 gene allele (as proxy for family history of dementia) causes study participation (selection into the analytic sample). These processes are shown by the SCC model in Figure 5B, where sufficient causes with low education/*Z*1, *Z*5, and APOE ε4/*Z*6 represent sufficient causes for dementia, and X1/high education, X2, and X3/APOE ε4 represent sufficient causes for study participation. Note that in the sufficient cause for study participation, high education (i.e., the *absence of* low education) with X1 causes study participation.

Both low education and APOE ε4 are exogenous in the DAG in Figure 5A, and therefore independent from one another in the full simulated sample. However, because low education is negatively associated with study participation but APOE ε4 is positively associated with study participation, among those in the analytic sample, conditioning on the collider will induce a positive association between low education and APOE ε4 (Figure 5A). Because APOE ε4 is a cause of dementia, a positive association is thus induced between low education and dementia *in the analytic sample,* even though there is no causal relationship between them in truth (Figure 5A) (24).

Again, for illustrative purposes, we simulated under the sharp null, setting Z1 to 0, making the true RR and RD 1 and 0, respectively (Section 3 of accompanying code). Other causal component prevalences were set to produce collider bias that yielded a crude association in the analytic sample of approximately 1.55 (Section 3A-B). In this scenario, the simulated sample of 10,000 individuals is not the same as the analytic sample (target population for our estimand, See Methods). Rather, the analytic sample comprises individuals who participate in the study; study participation itself is modeled as an endogenous variable in the simulation (i.e., determined by an individual's causal components, Figure 5B).

Across the simulations, the observed effects *in the full simulated sample of 10,000* were consistent with the true effects (RR_crude_ 1.00 [0.92–1.09] and RD_crude_ 0.000 [−0.016–0.016], code Section 3C). However, conditioning on study participation creates collider bias, such that the crude estimates *in the analytic sample* (mean *N* = 3,309) were inconsistent with the truth (RR_crude_ 1.55 [1.38–1.74] and RD_crude_ 0.117 [0.087–0.147]). If we have a measure of the collider-inducing variable (APOE ε4), we can employ standardization to correct for collider bias. After standardizing to the prevalence of APOE ε4 in the study sample, the sample RR and RD were consistent with the true effect (RR_adj_ 1.00 [0.90–1.12], RD_adj_ 0.00 [−0.029–0.031]). Again, as a companion to simulation results, Supplementary Appendix 3 includes detailed mathematical derivation of the crude and adjusted RR and RD values.

The mechanism by which the crude effect in the sample was biased is clearly shown by examination of response types in Table 4 (code Section 3D). In the full simulated sample of 10,000, exchangeability is met, as there are a similar proportion of “doomed” types in the exposed and unexposed. However, after conditioning on study participation (inclusion in the analytic sample), exchangeability fails: the “doomed” (those with APOE ε4 and Z6) are overrepresented in the exposed (low education) group, leading to the biased crude estimates. The reason for greater prevalence of doomed among exposed in the study sample is explained by how people enter the sample. In order to be in the study sample, participants need to have either high education and X1, or, if they have low education (our exposure of interest), they must complete either the sufficient cause X2 or APOE ε4/X3. As a result, although in the full simulated sample low education and APOE ε4 are unassociated, in the analytic sample a positive association between them is induced because those with low education (which increases likelihood of non-participation in the study) who end up in the study sample are more likely to have APOE ε4 (a cause of study participation) (Figure 5A). Because APOE ε4 is also a cause of dementia (with Z6), this results in overrepresentation of the doomed response types in the low education group in the study sample.

**Table 4:** SCC components and response types for simulations demonstrating collider bias.

	* *	In full simulated sample		In analytic sample	
Individual's response type (effect of low education on dementia)	Individual's characteristics (risk set type)^a^	Prevalence of RT in those with low education (*E*+)	Prevalence of RT in those with high education (*E*-)	Prevalence of RT in those with low education (*E*+)	Prevalence of RT in those with high education (*E*-)
Doomed	Z1_(1)_Z5_(1)_Z6_(1)_APOE4_(1)_ Z1_(0)_Z5_(1)_Z6_(1)_APOE4_(1)_ Z1_(1)_Z5_(1)_Z6_(1)_APOE4_(0)_ Z1_(0)_Z5_(1)_Z6_(1)_APOE4_(0)_ Z1_(1)_Z5_(1)_Z6_(0)_APOE4_(1)_ Z1_(0)_Z5_(1)_Z6_(0)_APOE4_(1)_ Z1_(1)_Z5_(1)_Z6_(0)_APOE4_(0)_ Z1_(0)_Z5_(1)_Z6_(0)_APOE4_(0)_ Z1_(1)_Z5_(0)_Z6_(1)_APOE4_(1)_ Z1_(0)_Z5_(0)_Z6_(1)_APOE4_(1)_	19.0% (18.1%–19.9%)	19.0% (17.7%–20.4%)	33.3% (30.9%–35.6%)	21.6% (19.7%–23.4%)
Causal^b^	Z1_(1)_Z5_(0)_Z6_(0)_APOE4_(0)_ Z1_(1)_Z5_(0)_Z6_(1)_APOE4_(0)_ Z1_(1)_Z5_(0)_Z6_(0)_APOE4_(1)_	0%	0%	0%	0%
Preventive	N/A	N/A	N/A	N/A	N/A
Immune	Z1_(0)_Z5_(0)_Z6_(0)_APOE4_(0)_ Z1_(0)_Z5_(0)_Z6_(1)_APOE4_(0)_ Z1_(0)_Z5_(0)_Z6_(0)_APOE4_(1)_	81.0% (80.1%–81.9%)	81.0% (79.6%–82.3%)	66.7% (64.4%–69.1%)	78.4% (76.6%–80.3%)

RT, response type; *E*+, exposed; *E*-, unexposed; N/A, not applicable.

^a^ Risk set notation: Z1_(1)_ indicates the individual has causal component Z1, while Z1_(0)_ indicates they do not have Z1.

^b^ In the simulation, Z1 was set to zero to simulate under the sharp null.

### Effect modification

**Figure 6:**
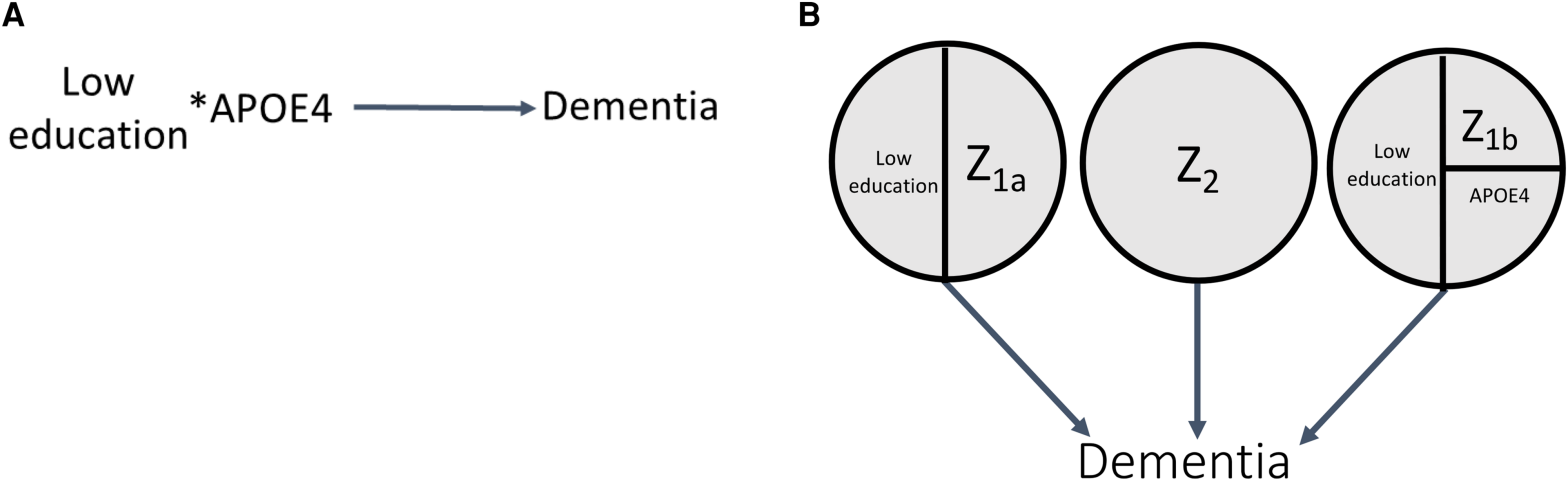
DAG and SCC model for effect modification.

Researchers are often interested in understanding whether the effect of an exposure depends on the level of another variable (e.g., effect modification) (4, 28). The SCC model is exceptionally well-suited for illustrating the causal mechanism for effect modification. For our education and dementia example, APOE ε4 status has also been proposed as a modifier of the effect of low education on dementia (29, 30). Although a number of approaches have been proposed to show effect modification in DAGs (31–34), there is no consistent standard in the field; we represent effect modification as shown in Figure 6A. Although all causal complements of the exposure of interest are effect modifiers in the SCC framework, when exploring effect modification we explicitly name the causal complement that is the effect modifier of interest, as shown in the SCC model in Figure 6B (i.e., in the right-most sufficient cause, APOE ε4 is the effect modifier of explicit interest and is expressed in the sufficient cause in addition to Z1b, which represents all other causal complements low education works with to cause dementia). This model articulates two mechanisms by which low education causes dementia, splitting the sufficient cause with low education/Z1 (used in previous examples) into two sufficient causes: in one, low education and Z1a are sufficient to produce dementia (i.e., in some individuals, low education does not have to work with APOE ε4 to cause dementia) and in the other, low education along with the modifier of interest APOE ε4 and Z1b are sufficient to produce dementia. The inclusion of Z1b indicates that low education and APOE ε4 are insufficient on their own to cause dementia.

In Section 4 of the companion code, we set the prevalences of Z1a, Z2, and Z1b to yield true causal effects of RR = 1.58 and RD = 0.058 (code Section 4A-B); we specified larger true effects (RR = 2.22, RD = 0.122) among those who carry the APOE ε4 allele, and smaller true effects (RR = 1.36, RD = 0.036) among those who do not. Because no bias was simulated in this scenario, crude effects across the simulated samples closely mirrored these true effects (code Section 4C). The overall RR was 1.58 (95% CI 1.41–1.78), and RD was 0.058 (0.044–0.071); stratified results were RR = 2.26 (1.79–2.86), RD = 0.122 (0.094–0.153) among those with APOE ε4, and RR = 1.36 (1.19–1.56), RD = 0.036 (0.021–0.051) among those without it. Supplementary Appendix 4 includes mathematical derivations of the crude RR and RD values in the total sample and within strata of APOE ε4, as a companion to the simulation code.

There are two important concepts illustrated by the simulation results shown in Table 5 (code Section 4D). First, across the simulated samples, there is exchangeability (balanced distributions of response types between exposed and unexposed) in the sample overall, and within strata of APOE ε4. This is helpful in clarifying for students that effect modification is a causal process, rather than a source of bias. Second, the proportion of causal response types is larger in the stratum with APOE ε4 (12.2% vs. 3.6%). This occurs because in this simulation, we specifically articulated two mechanisms underlying the causal effect of low education on dementia. In the stratum without APOE ε4, nobody can complete the sufficient cause with low education, APOE ε4, and Z1b, limiting the proportion of causal response types in this stratum. In the APOE ε4+ stratum, this sufficient cause can be completed by individuals with Z1b, increasing the proportion of causal response types in this stratum. Thus, the SCC simulations help students “see” the causal mechanism driving different effect sizes between strata of the modifier.

**Table 5:** SCC components and response types for simulations demonstrating effect modification.

Overall			
**Individual's response type (effect of low education on dementia)**	**Individual's characteristics (risk set type)^a^**	**Prevalence of RT in those with low education (*E*+)**	**Prevalence of RT in those with high education (*E*-)**
Doomed	Z1a_(1)_Z2_(1)_Z1b_(1)_APOE4_(1)_ Z1a_(0)_Z2_(1)_Z1b_(1)_APOE4_(1)_ Z1a_(1)_Z2_(1)_Z1b_(0)_APOE4_(1)_ Z1a_(0)_Z2_(1)_Z1b_(0)_APOE4_(1)_ Z1a_(1)_Z2_(1)_Z1b_(1)_APOE4_(0)_ Z1a_(0)_Z2_(1)_Z1b_(1)_APOE4_(0)_ Z1a_(1)_Z2_(1)_Z1b_(0)_APOE4_(0)_ Z1a_(0)_Z2_(1)_Z1b_(0)_APOE4_(0)_	10.0% (9.3%–10.7%)	10.0% (9.0%–11.0%)
Causal	Z1a_(1)_Z2_(0)_Z1b_(0)_APOE4_(1)_ Z1a_(1)_Z2_(0)_Z1b_(1)_APOE4_(1)_ Z1a_(0)_Z2_(0)_Z1b_(1)_APOE4_(1)_ Z1a_(1)_Z2_(0)_Z1b_(0)_APOE4_(0)_ Z1a_(1)_Z2_(0)_Z1b_(1)_APOE4_(0)_	5.8% (5.3%–6.3%)	5.8% (5.0%–6.6%)
Preventive	N/A	N/A	N/A
Immune	Z1a_(0)_Z2_(0)_Z1b_(0)_APOE4_(1)_ Z1a_(0)_Z2_(0)_Z1b_(0)_APOE4_(0)_ Z1a_(0)_Z2_(0)_Z1b_(1)_APOE4_(0)_	84.2.% (83.3%–85.1%)	84.2% (82.9%–85.5%)
Among those *with* APOE ε4			
**Individual's response type (effect of low education on dementia)**	**Individual's characteristics**	**Prevalence of RT in those with low education (*E*+)**	**Prevalence of RT in those with high education (*E*-)**
Doomed	Z1a_(1)_Z2_(1)_Z1b_(1)_APOE4_(1)_ Z1a_(0)_Z2_(1)_Z1b_(1)_APOE4_(1)_ Z1a_(1)_Z2_(1)_Z1b_(0)_APOE4_(1)_ Z1a_(0)_Z2_(1)_Z1b_(0)_APOE4_(1)_	10.0% (8.6%–11.5%)	10.0% (7.8%–12.2%)
Causal	Z1a_(1)_Z2_(0)_Z1b_(0)_APOE4_(1)_ Z1a_(1)_Z2_(0)_Z1b_(1)_APOE4_(1)_ Z1a_(0)_Z2_(0)_Z1b_(1)_APOE4_(1)_	12.2% (10.7%–12.7%)	12.3% (10.2%–14.5%)
Preventive	N/A	NA	NA
Immune	Z1a_(0)_Z2_(0)_Z1b_(0)_APOE4_(1)_	77.7% (76.0%–79.6%)	77.7% (74.7%–80.5%)
Among those *without* APOE ε4			
**Individual's response type (effect of low education on dementia)**	**Individual's characteristics**	**Prevalence of RT in those with low education (*E*+)**	**Prevalence of RT in those with high education (*E*-)**
Doomed	Z1a_(1)_Z2_(1)_Z1b_(1)_APOE4_(0)_ Z1a_(0)_Z2_(1)_Z1b_(1)_APOE4_(0)_ Z1a_(1)_Z2_(1)_Z1b_(0)_APOE4_(0)_ Z1a_(0)_Z2_(1)_Z1b_(0)_APOE4_(0)_	10.0% (9.3%–10.8%)	10.0% (8.8%–11.2%)
Causal	Z1a_(1)_Z2_(0)_Z1b_(0)_APOE4_(0)_ Z1a_(1)_Z2_(0)_Z1b_(1)_APOE4_(0)_	3.6% (3.1%–4.1%)	3.6% (2.9%–4.4%)
Preventive	N/A	NA	NA
Immune	Z1a_(0)_Z2_(0)_Z1b_(0)_APOE4_(0)_ Z1a_(0)_Z2_(0)_Z1b_(1)_APOE4_(0)_	86.4% (85.5%–87.3%)	86.4% (85.0%–87.7%)

RT, response type; *E*+, exposed; *E*-, unexposed; N/A, not applicable.

^a^ Risk set notation: Z1a_(1)_ indicates the individual has causal component Z1a, while Z1a_(0)_ indicates they do not have Z1.a.

We note that researchers could additionally be interested in the causal effects of APOE ε4 in strata of education (4, 28). For this research question, the number of response types expands to include all combinations of exposure levels for both low education and APOE ε4, beyond the scope of this introductory paper, but work examining the correspondence between SCC components and interaction response types has been published elsewhere (4, 7, 35).

## Discussion and conclusions

Simulation is a useful tool for teaching causal inference in epidemiologic training. In this paper, we have summarized simulations based in the SCC framework, and provided simple examples and code to illustrate how these simulations may help students better understand four common causal structures, including both causal mechanisms and sources of bias. Specifically, these examples may help students better understand how different sources of bias arise, their consequences for effect estimation, and how to correct for them.

Most didactic papers on simulations or simulation tutorials use regression-based methods for generating data that simulate directly from DAGs (1, 36, 37). A key benefit to SCC simulations is that, in addition to being consistent with the heuristic used to teach students about causation, they enable students to “see” each individual's response type, thereby revealing the mechanisms by which bias (i.e., non-exchangeability) occurred. As noted previously, an individual's response type is determined by their distribution of component causes other than the exposure of interest (i.e., their “risk set type”). By simulating all component causes for each simulated individual, that person's risk set type, and therefore response type, is known, regardless of their actual exposure and outcome. In contrast, in regression-based simulations, only a simulated individual's actual exposure and actual outcome are known (similar to in empirical data). Although the truth, observed estimate, and bias are known in both types of simulations, only SCC simulations can show underlying response types and non-exchangeability that lead to the observed estimate, helping students to gain deeper understanding of how a particular causal structure leads to a corresponding pattern of observed data.

Although one could design a data generating mechanism and simulation exclusively using the SCC model, we include translation from DAGs to SCC models as a first step to our approach. DAGs are a familiar and predominant tool in epidemiologic teaching that introduce students to the idea of bias resulting from backdoor paths. However, while DAGs help visualize the existence of biasing pathways, they do not necessarily help show *why* bias arises. Used in tandem, we believe DAGs and SCC-based simulations reinforce each other and enhance students' understanding of how different biases arise and their consequences; for example, while a DAG can illustrate confounding, alerting students to the existence of a backdoor path, the SCC simulation *shows* the consequences of confounding as the imbalance of other causes of disease (i.e., doomed response types) among the exposed and unexposed groups in the sample.

There are some important considerations for using SCC simulations in the teaching setting. To function as useful pedagogic examples, our simulations made some simplifying assumptions (e.g., monotonicity, binary variables, and in some cases, simulating under the sharp null). While our examples do not reflect the complexity of real-world data generating mechanisms, their simplicity makes for straightforward illustration of key concepts from each scenario and the differences between scenarios, a common tradeoff made in teaching settings and methodological simulation work. It should be noted that relaxing these assumptions presents no conceptual barrier to our approach. For example, we also provide results (Supplementary Appendix 2) and example code (last section of Supplementary Appendix 5) for a confounding simulation not under the sharp null. In addition, although the SCC framework requires binary variables (6), the simulations we present can be adapted to accommodate non-binary variables using indicator variables to represent levels of categorical variables or ranges of continuous variables in sufficient causes. However, practically, more complex SCC models increases the number of sets of sufficient causes and the relationships between them. As a result, because specification of the true effects in an SCC simulation is based on the distribution of component causes rather than a single beta coefficient, increasing complexity of the SCC model complicates tasks like choosing prevalences of component causes to achieve a desired effect size (as shown in Supplementary Appendix 1) or computing expected values for RRs and RDs to compare with simulation results (as done in Supplementary Appendix 1–4). We believe such complications are often unnecessary, and in fact may hinder the goal of using SCC simulations to help students gain deeper understanding and an intuition regarding how common causal structures produce statistical associations, and mechanisms through which bias arises.

We anticipate that the best way to incorporate SCC simulations into epidemiologic teaching depends on the level of the students and teaching goals. For all students, prior knowledge of potential outcomes, DAGs, and some exposure to the SCC framework will be a useful starting point. For students very early in epidemiologic training or with little exposure to coding and data analysis, the optimal use of SCC simulations may be for instructors to generate teaching examples, and focus on walking through the figures and tables for each causal structure. For more advanced students with some coding and data analysis experience, instructors could provide code to generate data, and ask students to generate summaries of response types and effect estimates. Instructors may tailor the applied examples, code, and causal structures of interest (e.g., a DAG with both confounding and collider bias to show additional complexity that may be present in empirical data) according to their substantive expertise, course level, and learning objectives. Broadly, we anticipate that incorporation of SCC simulations into epidemiologic coursework will help students build deeper understanding about *how* certain causal structures lead to observed associations, which will in turn help them build intuition about what they can expect to see and forms of bias they will likely encounter when conducting analyses in empirical data.

Overall, we believe SCC-based simulations are an underutilized resource in epidemiologic teaching, and a useful tool for building intuition about causal mechanisms and bias. SCC simulations enable students to understand and visualize the mechanisms through which causation and bias occur on an individual level. In this article, we have outlined the process and key considerations for simulating from the SCC framework, and hope that our illustrative examples and sample R code facilitate their greater use as teaching tools.

## Data Availability

The original contributions presented in the study are included in the article/**Supplementary Material**, further inquiries can be directed to the corresponding author.

## Supplementary material

The supplementary file Supplementary Appendix 5 “SCC sims companion code.R” contains the code to generate each set of simulation results in the paper. It is also available on the authors' github: (https://github.com/ehayeslarson/SCCsims). Appendix 1 through Appendix 4 provide mathematical computation of crude and standardized effect measures for all simulation scenarios, to accompany the simulation code/results and suit different learning styles.
