# Supplementary material for: Sufficient component cause simulations: an underutilized epidemiologic teaching tool
Source: Front Epidemiol. 2023 Nov 10;3:1282809. doi: 10.3389/fepid.2023.1282809 (PMC10906966; doi:10.3389/fepid.2023.1282809)
Supplement: Supplementary file 1 [file Datasheet1.zip › Appendix 1. Causation.DOCX]

**Appendix 1. Causation**

| **DAG** | **SCC model equivalent of DAG** | **Parameter inputs**  **from simulation** | **True effects** |
| --- | --- | --- | --- |
| 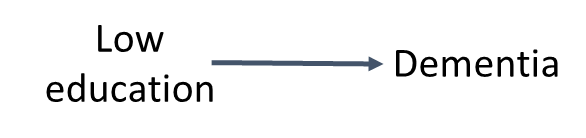 | 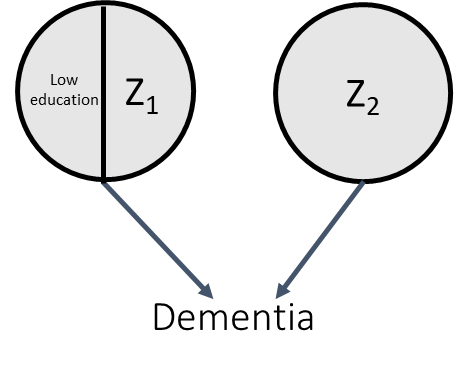 | Prevalence of exogenous variables from simulation:  pZ1=0.06  pZ2=0.1  pLowEd=0.7 | RR = 1.54  RD = 0.054 |

In the causation example, because low education (the exposure) is independent of other variables, the crude risk ratio and difference are equal to the causal quantities (i.e., true effects) specified in the simulation. They can be computed as follows:

$${Risk ratio}_{crude}= \frac{P (Dementia|Low Education)}{P (Dementia|\bar{Low Education})}$$

${Risk difference}_{crude}=$ $P (Dementia|Low Education)-P (Dementia|\bar{Low Education}$)

The numerator and denominator quantities can be derived from the relevant causal components in Figure 3B and their parameter values specified in the simulation. For example, given an individual has low education, they will get dementia if they have either components Z1 or Z2. Likewise, given an individual does not have low education, they will get dementia only if they have component Z2. The prevalences of Z1 and Z2 are specified above as 0.06 and 0.10, respectively.

*Thus*:

$P (Dementia|Low Education)$ = $P((Z_{1}\cup Z_{2})|Low Education)$ = $P\left( Z_{1}\cup Z_{2} \right), because Z_{1}, Z_{2}\perp Low Education$

= ${P(Z}_{1})+{P(Z}_{2})-{P(Z}_{1}){*P(Z}_{2})$

*note, the intersection is subtracted to account for redundancy i.e., a person completing multiple sufficient causes by the end of the study period when their outcome status is ascertained*

= 0.06 + 0.10 – (0.1*0.06)

= 0.154

$P (Dementia|\bar{Low Education)}$ = ${P(Z}_{2}\left| \bar{Low Education} \right)=P\left( Z_{2} \right), because Z_{2}\perp\bar{Low Education}$

= 0.1

*And*:

${Risk ratio}_{crude} =\frac{0.154}{0.1}=1.54$

${Risk difference}_{crude}= 0.154-0.1=0.054$

Again, the values for the RR and RD obtained here are unbiased (i.e., equal to the true effects specified in the simulation) because we created a data generating mechanism where Low Education is independent of other variables in the sample. Thus, we expect there to be exchangeability between the exposed and unexposed on other causes of disease (i.e., response types). Consistent with this, in Table 2 we see that in our simulations, the population prevalence of doomed response types is 0.10 among individuals exposed and unexposed to Low Education.

We note that when specifying the parameters of the simulation, if a certain effect size is desired it can be derived by setting a value for, say, the RR and then either setting P(Z1) or P(Z2) (i.e., parameters pZ1 and pZ2 in the simulation code, respectively) to a fixed value and solving for the other. For example, if an RR of 2.5 was desired and the prevalence of all other causes of some outcome (i.e., pZ2, or the prevalence of the outcome in the unexposed) was assumed to be 15%, then the input value of the simulation parameter pZ1 should be specified as 0.2647.

i.e., RR = ${(P(Z}_{1})+{P(Z}_{2})-P(Z_{1}){*P(Z}_{2})$) / P($Z_{2})$

2.5 = ${(P(Z}_{1})+0.15-{P(Z}_{1})*0.15)$ / $0.15$

0.375 = ${(P(Z}_{1})+0.15-P(Z_{1})*0.15)$

0.225 = ${0.85*P(Z}_{1})$

0.2647 $= {P(Z}_{1})=pZ1$

As the complexity of the data generating mechanism increases, so do the formulae for determining the values of various components to achieve a desired effect size. However, for these simple “toy” examples used for pedagogic purposes, this procedure is fairly straightforward. Instructors wishing to use other substantive examples, or desiring to specifically demonstrate other concepts (e.g. rare vs. common disease outcome) can choose prevalences of causal components accordingly.
