# Supplementary material for: Sufficient component cause simulations: an underutilized epidemiologic teaching tool
Source: Front Epidemiol. 2023 Nov 10;3:1282809. doi: 10.3389/fepid.2023.1282809 (PMC10906966; doi:10.3389/fepid.2023.1282809)
Supplement: Supplementary file 1 [file Datasheet1.zip › Appendix 4. Effect modification proof.DOCX]

**Appendix 4. Effect modification proof**

| **DAG** | **SCC model equivalent of DAG** | **Parameter inputs from simulation** | **True**  **effects** |
| --- | --- | --- | --- |
| 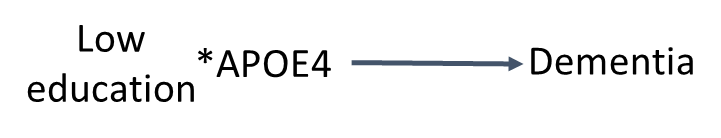 |  | Prevalence of exogenous variables in the simulation:  pZ1a=0.04  pZ2=0.1  pZ1b=0.1  pAPOE4=0.25  pLowEd=0.7 | Total sample:  RR=1.58  RD=0.058  APOE4+ stratum  RR=2.22  RD=0.122  APOE4- stratum  RR=1.36  RD=0.036 |

**I. Estimates in the total sample**

We first compute the crude risk ratio and risk difference in the total sample as follows:

$${Risk ratio}_{crude}= \frac{P (Dementia|Low Education)}{P (Dementia|\bar{Low Education})}$$

$R{isk difference}_{crude}=$ $P (Dementia|Low Education)-P (Dementia|\bar{Low Education}$)

The numerator and denominator quantities can be derived from the relevant causal components in Figure 6B and their parameter values specified in the simulation. For example, given an individual has low education, they will get dementia if they have either components Z_1a_, Z_2_, or APOE4&Z_1b_. Likewise, given an individual does not have low education, they will get dementia only if they have component Z_2_. The values of these component causes are specified above.

*Thus:*

$P (Dementia|Low Education)$ = ${P((Z}_{1a}\cup Z_{2}$ $\cup{(APOE4\cap Z}_{1b}))|Low Education$)

= ${P(Z}_{1a}\cup Z_{2}$ $\cup{(APOE4\cap Z}_{1b})),$ $because Z_{1a}, Z_{2},APOE4, Z_{1b}\perp Low Education$

= 0.04+0.1+(0.25*0.1)–(0.04*0.1)–(0.04*0.25*0.1)–(0.1*0.25*0.1)+(0.04*0.1*0.25*0.1)

= 0.1576

$P (Dementia|\bar{Low Education})$ = ${P(Z}_{2}\left| \bar{Low Education} \right)=P\left( Z_{2} \right), because Z_{2}\perp\bar{Low Education}$

= 0.1

*And:*

$Risk ratio=\frac{0.1576}{0.1}=1.576 \sim1.58$

$Risk difference= 0.1576-0.1=0.0576 \sim0.058$

These values for the crude RR and RD are unbiased (i.e., equal to the true effects specified in the simulation); the crude estimate is unbiased because of exchangeability of response types between exposed and unexposed, as shown in the main text Table 5.

**II. Estimates within strata of the effect modifier, APOE4**

To compute the crude risk ratio and risk difference among those with and without APOE4, we follow the same procedures as in Part I, only we condition on having and not having APOE4.

*Among those with APOE4:*

$P (Dementia|Low Education, APOE4)$ = $P(Z_{1a}\cup Z_{2}$ $\cup Z_{1b}|Low Education, APOE4)$

$=P(Z_{1a}\cup Z_{2}$ $\cup Z_{1b})$, ${because Z}_{1a}, Z_{2}, Z_{1b}\perp Low Education$

${and Z}_{1a}, Z_{2}, Z_{1b}\perp APOE4$

= 0.04 + 0.1 + 0.1 – (0.04*0.1) – (0.04*0.1) – (0.1*0.1) + (0.04*0.1*0.1)

= 0.2224

$P (Dementia|\bar{Low Education}$, APOE4) = ${P(Z}_{2}\left| \bar{Low Education},APOE4 \right)$

$=P\left( Z_{2} \right), because Z_{2}\perp\bar{Low Education}$ and $Z_{2}\perp$ $APOE4$

= 0.1

*Among those without APOE4:*

$P \left( Dementia | Low Education, \bar{APOE4} \right)$ = $P\left( Z_{1a}\cup Z_{2} | Low Education, \bar{APOE4} \right)$

$$={P(Z}_{1a}\cup Z_{2}), because {Z_{1a},Z}_{2}\perp Low Education$$

$$and {Z_{1a},Z}_{2}\perp\bar{APOE4}$$

= 0.04 + 0.1 - (0.04*0.1)

= 0.136

$P (Dementia|\bar{Low Education}$, $\bar{APOE4}$) = ${P(Z}_{2}\left| \bar{Low Education}, \bar{APOE4} \right)$

$$=P\left( Z_{2} \right), because Z_{2}\perp\bar{Low Education}$$

$$and Z_{2}\perp\bar{APOE4}$$

= 0.1

Again, the values for the RR and RD obtained within strata of APOE ε4 are unbiased (i.e., equal to the true effects specified in the simulation). As with the overall sample, there is exchangeability between exposed and unexposed within strata of APOE ε4, as shown in Table 5 in the main text. While possible to compute from the formulas above, Table 5 also more easily shows the proportions of doomed and causal types in each strata of exposure and APOE ε4, showing the higher prevalence of dementia in the exposed in the APOE ε4+ strata relative to the exposed in the APOE ε4- strata is due to a larger proportion of causal response types.
