# Supplementary material for: Sufficient component cause simulations: an underutilized epidemiologic teaching tool
Source: Front Epidemiol. 2023 Nov 10;3:1282809. doi: 10.3389/fepid.2023.1282809 (PMC10906966; doi:10.3389/fepid.2023.1282809)
Supplement: Supplementary file 1 [file Datasheet1.zip › Appendix 5. Companion code.DOCX]

**Appendix 5. Companion code**

####################################################

##Section 1: Simulating causal effect

####################################################

niterations<-1000

blanks<-rep(NA,niterations)

results_CE<-data.frame(TrueRR=blanks, TrueRD=blanks,

CrudeRR=blanks, CrudeRD=blanks,

Doomed_E1=blanks, Causal_E1=blanks, Immune_E1=blanks,

Doomed_E0=blanks, Causal_E0=blanks, Immune_E0=blanks)

#Section 1A: Set prevalence of exogenous variables

pZ1<-0.06

pZ2<-0.1

pLowEd<-0.7 #the exposure ("E" in the formulas below)

#Section 1B: Simulate cohort of n=10,000 individuals

sampsize<-10000

set.seed(12345) #set a seed so that results can be replicated

for (i in 1:niterations){

#Simulate exogeneous variables

df<-data.frame(ID=seq(1,sampsize),

Z1=rbinom(sampsize,1,pZ1),

Z2=rbinom(sampsize,1,pZ2),

LowEd=rbinom(sampsize,1,pLowEd),

Dementia=NA)

#Simulate endogenous variables based on SCCs

df$Dementia<-ifelse((df$LowEd==1 & df$Z1==1) | df$Z2==1,1,0) #whether an individual has dementia or not (the outcome, "Y") depends on their value of causal components in the pies whose completion leads to dementia

#Section 1C: Calculate effects

pY_E0<-sum(df$LowEd==0 & df$Dementia==1)/sum(df$LowEd==0)

pY_E1<-sum(df$LowEd==1 & df$Dementia==1)/sum(df$LowEd==1)

CrudeRR<-pY_E1/pY_E0

CrudeRD<-pY_E1-pY_E0

TrueRR <- (pZ1+pZ2-pZ1*pZ2)/pZ2

TrueRD <- (pZ1+pZ2-pZ1*pZ2)-pZ2

#For causation scenario we expect Crude measures = True measures because there are no sources of non-exchangeability

#Section 1D: "Under the hood" (define response types based on SCCs)

df$RT<-ifelse(df$Z2==1,"Doomed",ifelse(df$Z1==1, "Causal","Immune") )

RTtab<- t(table(df$LowEd,df$RT)/c(sum(df$LowEd==0),sum(df$LowEd==1)))

RTtab

#Store results

results_CE$TrueRR[i]<-TrueRR

results_CE$TrueRD[i]<-TrueRD

results_CE$CrudeRR[i]<-CrudeRR

results_CE$CrudeRD[i]<-CrudeRD

results_CE$Doomed_E1[i]<-RTtab["Doomed","1"]

results_CE$Doomed_E0[i]<-RTtab["Doomed","0"]

results_CE$Causal_E1[i]<-RTtab["Causal","1"]

results_CE$Causal_E0[i]<-RTtab["Causal","0"]

results_CE$Immune_E1[i]<-RTtab["Immune","1"]

results_CE$Immune_E0[i]<-RTtab["Immune","0"]

}

#Calculate 95% CI

mean_CIs<-function(data, estimate){

results<-data.frame(Est=estimate)

results$LCI<-quantile(data[,estimate],probs=.025)

results$mean<-mean(data[,estimate])

results$UCI<-quantile(data[,estimate],probs=.975)

return(results)

}

#Auto-generate a results table

for (i in 1:length(colnames(results_CE))){

if (i==1){

res_tab_CE<-mean_CIs(results_CE,colnames(results_CE)[i])

} else{

res_tab_CE<-rbind(res_tab_CE, mean_CIs(results_CE,colnames(results_CE)[i]))}

}

#Print table of results:

#Average true and crude RRs and RDs across simulations and their 95% CI

#Average prevalence of response types within exposure groups across simulations and their 95% CI

res_tab_CE

#Drop all objects except results tables

rm(list=grep("res_tab",ls(),value=TRUE,invert=TRUE))

####################################################

##Section 2: Simulating confounding

####################################################

niterations<-1000

blanks<-rep(NA,niterations)

results_conf<-data.frame(TrueRR=blanks, TrueRD=blanks,

CrudeRR=blanks, CrudeRD=blanks,

AdjRR=blanks, AdjRD=blanks,

Doomed_E1=blanks,Immune_E1=blanks,

Doomed_E0=blanks,Immune_E0=blanks)

#Section 2A: Set prevalence of exogenous variables

pQ1<-0.8

pQ2<-0.2

pZ1<-0 #setting the causal partner of the exposure to 0 results in a null effect

pZ3<-0.1

pZ4<-0.15

pLowSES<-0.7

#Section 2B: Simulate cohort of n=10,000 individuals

sampsize<-10000

set.seed(12345) #set a seed so that results can be replicated

for (i in 1:niterations){

#Simulate exogeneous variables

df<-data.frame(ID=seq(1,sampsize),

Q1=rbinom(sampsize,1,pQ1),

Q2=rbinom(sampsize,1,pQ2),

LowSES=rbinom(sampsize,1,pLowSES),

Z1=rbinom(sampsize,1,pZ1),

Z3=rbinom(sampsize,1,pZ3),

Z4=rbinom(sampsize,1,pZ4),

LowEd=NA,

Dementia=NA)

#Simulate endogenous variables based on SCCs

df$LowEd<-ifelse((df$LowSES==1 & df$Q1==1) | df$Q2==1,1,0) #in Scenario 2 (confounding), the exposure (LowEd or "E" in the formulas below) is no longer exogenous; each individual's value for LowEd emerges from the values of the components that cause it

df$Dementia<-ifelse((df$LowEd==1 & df$Z1==1) | df$Z3==1 | (df$LowSES==1 & df$Z4==1),1,0) #whether an individual has dementia or not (the outcome, "Y") depends on their value of causal components in the pies whose completion leads to dementia

#Section 2C: Calculate effects

#Truth (true ATE)

TrueRR<-(pZ1+pZ3+pLowSES*pZ4-pZ1*pZ3-pZ1*pLowSES*pZ4-pZ3*pLowSES*pZ4-pZ1*pZ3*pLowSES*pZ4)/(pZ3+pLowSES*pZ4-pZ3*pLowSES*pZ4)

TrueRD<-(pZ1+pZ3+pLowSES*pZ4-pZ1*pZ3-pZ1*pLowSES*pZ4-pZ3*pLowSES*pZ4-pZ1*pZ3*pLowSES*pZ4)-(pZ3+pLowSES*pZ4-pZ3*pLowSES*pZ4)

#Crude estimates

pY_E0<-sum(df$LowEd==0 & df$Dementia==1)/sum(df$LowEd==0)

pY_E1<-sum(df$LowEd==1 & df$Dementia==1)/sum(df$LowEd==1)

CrudeRR<-pY_E1/pY_E0

CrudeRD<-pY_E1-pY_E0

CrudeRR

CrudeRD

#Standardized (adjusted) estimates

pY_E0_C0<-sum(df$LowEd==0 & df$LowSES==0 & df$Dementia==1)/sum(df$LowEd==0 & df$LowSES==0)

pY_E0_C1<-sum(df$LowEd==0 & df$LowSES==1 & df$Dementia==1)/sum(df$LowEd==0 & df$LowSES==1)

pY_E1_C0<-sum(df$LowEd==1 & df$LowSES==0 & df$Dementia==1)/sum(df$LowEd==1 & df$LowSES==0)

pY_E1_C1<-sum(df$LowEd==1 & df$LowSES==1 & df$Dementia==1)/sum(df$LowEd==1 & df$LowSES==1)

pC1<-nrow(df[df$LowSES==1,])/nrow(df)

pC0<-1-pC1

pY_E1_std<-pY_E1_C1*pC1+pY_E1_C0*pC0

pY_E0_std<-pY_E0_C1*pC1+pY_E0_C0*pC0

AdjRR <- pY_E1_std/pY_E0_std

AdjRD <- pY_E1_std-pY_E0_std

AdjRR

AdjRD

#Section 2D: "Under the hood" (define response types based on SCCs)

df$RT<-ifelse((df$Z3==1 | (df$LowSES==1 & df$Z4==1 )),"Doomed", ifelse(df$Z1==1, "Causal","Immune") )

RTtab<-t(table(df$LowEd,df$RT)/c(sum(df$LowEd==0),sum(df$LowEd==1)))

RTtab

#Store results

results_conf$TrueRR[i]<-TrueRR

results_conf$TrueRD[i]<-TrueRD

results_conf$CrudeRR[i]<-CrudeRR

results_conf$CrudeRD[i]<-CrudeRD

results_conf$AdjRR[i]<-AdjRR

results_conf$AdjRD[i]<-AdjRD

results_conf$Doomed_E1[i]<-RTtab["Doomed","1"]

results_conf$Doomed_E0[i]<-RTtab["Doomed","0"]

results_conf$Immune_E1[i]<-RTtab["Immune","1"]

results_conf$Immune_E0[i]<-RTtab["Immune","0"]

}

#Calculate 95% CI

mean_CIs<-function(data, estimate){

results<-data.frame(Est=estimate)

results$LCI<-quantile(data[,estimate],probs=.025)

results$mean<-mean(data[,estimate])

results$UCI<-quantile(data[,estimate],probs=.975)

return(results)

}

#Auto-generate a results table

for (i in 1:length(colnames(results_conf))){

if (i==1){

res_tab_conf<-mean_CIs(results_conf,colnames(results_conf)[i])

} else{

res_tab_conf<-rbind(res_tab_conf, mean_CIs(results_conf,colnames(results_conf)[i]))}

}

#Print table of results:

#Average true, crude, and adjusted RRs and RDs across simulations and their 95% CI

#Average prevalence of response types within exposure groups across simulations and their 95% CI (no causal types because simulating under the null)

res_tab_conf

#Drop all objects except results tables

rm(list=grep("res_tab",ls(),value=TRUE,invert=TRUE))

####################################################

##Section 3: Simulating collider bias

####################################################

niterations<-1000

blanks<-rep(NA,niterations)

results_coll<-data.frame(

TrueRRpop=blanks, TrueRDpop=blanks,

CrudeRRpop=blanks, CrudeRDpop=blanks,

CrudeRRsamp=blanks, CrudeRDsamp=blanks,

AdjRRsamp=blanks, AdjRDsamp=blanks,

Doomed_E1pop=blanks,Immune_E1pop=blanks,

Doomed_E0pop=blanks,Immune_E0pop=blanks,

Doomed_E1samp=blanks,Immune_E1samp=blanks,

Doomed_E0samp=blanks,Immune_E0samp=blanks,

nstudy=blanks)

#Section 3A: Set prevalence of exogenous variables

pX1<-0.5

pX2<-0.1

pX3<-0.5

pZ1<-0 #setting the causal partner of the exposure to 0 results in a null effect

pZ5<-0.1

pZ6<-0.4

pAPOE4<-0.25 #APOE4 is a cause of selection into the study sample, this is prevalence in the population

pLowEd<-0.7 #the exposure ("E" in the formulas below) is also a cause of being selected into the study sample

#Section 3B: Simulate cohort of n=10,000 individuals

sampsize<-10000

set.seed(12345) #set a seed so that results can be replicated

for (i in 1:niterations) {

#Simulate exogeneous variables

df<-data.frame(ID=seq(1,sampsize),

X1=rbinom(sampsize,1,pX1),

X2=rbinom(sampsize,1,pX2),

X3=rbinom(sampsize,1,pX3),

APOE4=rbinom(sampsize,1,pAPOE4),

Z1=rbinom(sampsize,1,pZ1),

Z5=rbinom(sampsize,1,pZ5),

Z6=rbinom(sampsize,1,pZ6),

LowEd=rbinom(sampsize,1,pLowEd),

StudyPart=NA,

Dementia=NA)

#Simulate endogenous variables based on SCCs

df$StudyPart<-ifelse((df$LowEd==0 & df$X1==1) | df$X2==1 | (df$APOE4==1 & df$X3==1),1,0) #Recall that high education (i.e., LowEd==0) is a cause of study participation

df$Dementia<-ifelse((df$LowEd==1 & df$Z1==1) | df$Z5==1 | (df$APOE4==1 & df$Z6==1),1,0)

#Section 3C: Calculate effects

#Truth (true ATE)

TrueRRpop<-(pZ1+pZ5+pAPOE4*pZ6-pZ1*pZ5-pZ1*pAPOE4*pZ6-pZ5*pAPOE4*pZ6-pZ1*pZ5*pAPOE4*pZ6)/(pZ5+pAPOE4*pZ6-pZ5*pAPOE4*pZ6)

TrueRDpop<-(pZ1+pZ5+pAPOE4*pZ6-pZ1*pZ5-pZ1*pAPOE4*pZ6-pZ5*pAPOE4*pZ6-pZ1*pZ5*pAPOE4*pZ6)-(pZ5+pAPOE4*pZ6-pZ5*pAPOE4*pZ6)

#With selection bias, we want to compare estimates in the whole population with estimates among the selected sample (our study participants)

#In whole population (dataframe "df")

pY_E0<-sum(df$LowEd==0 & df$Dementia==1)/sum(df$LowEd==0)

pY_E1<-sum(df$LowEd==1 & df$Dementia==1)/sum(df$LowEd==1)

#Crude estimates in whole population

CrudeRRpop<-pY_E1/pY_E0

CrudeRDpop<-pY_E1-pY_E0

CrudeRRpop

CrudeRDpop

#In those included in study sample (create new dataframe, "df_study")

df_study<-df[df$StudyPart==1,]

pY_E0_Study<-sum(df_study$LowEd==0 & df_study$Dementia==1)/sum(df_study$LowEd==0)

pY_E1_Study<-sum(df_study$LowEd==1 & df_study$Dementia==1)/sum(df_study$LowEd==1)

#Crude estimates in study sample

CrudeRR_Study<-pY_E1_Study/pY_E0_Study

CrudeRD_Study<-pY_E1_Study-pY_E0_Study

CrudeRR_Study

CrudeRD_Study

#Standardized (adjusted) estimates: Need to correct estimate in study sample with standardization for APOE4

pY_E0<-sum(df_study$LowEd==0 & df_study$Dementia==1)/sum(df_study$LowEd==0)

pY_E1<-sum(df_study$LowEd==1 & df_study$Dementia==1)/sum(df_study$LowEd==1)

pY_E0_C0<-sum(df_study$LowEd==0 & df_study$APOE4==0 & df_study$Dementia==1)/sum(df_study$LowEd==0 & df_study$APOE4==0)

pY_E0_C1<-sum(df_study$LowEd==0 & df_study$APOE4==1 & df_study$Dementia==1)/sum(df_study$LowEd==0 & df_study$APOE4==1)

pY_E1_C0<-sum(df_study$LowEd==1 & df_study$APOE4==0 & df_study$Dementia==1)/sum(df_study$LowEd==1 & df_study$APOE4==0)

pY_E1_C1<-sum(df_study$LowEd==1 & df_study$APOE4==1 & df_study$Dementia==1)/sum(df_study$LowEd==1 & df_study$APOE4==1)

sum(df_study$LowEd==1 & df_study$Dementia==1)/sum(df_study$LowEd==1)

sum(df_study$LowEd==0 & df_study$Dementia==1)/sum(df_study$LowEd==0)

pC1<-nrow(df_study[df_study$APOE4==1,])/nrow(df_study)

pC0<-1-pC1

pY_E1_std<-pY_E1_C1*pC1+pY_E1_C0*pC0

pY_E0_std<-pY_E0_C1*pC1+pY_E0_C0*pC0

AdjRR_Study <- pY_E1_std/pY_E0_std

AdjRD_Study <- pY_E1_std-pY_E0_std

AdjRR_Study

AdjRD_Study

#Section 3D: "Under the hood" (define response types based on SCCs)

df$RT<-ifelse((df$Z5==1 | (df$APOE4==1 & df$Z6==1 )),"Doomed", ifelse(df$Z1==1, "Causal","Immune") )

#Dist of RT in population

RTtabpop<-t(table(df$LowEd,df$RT)/c(sum(df$LowEd==0),sum(df$LowEd==1)))

RTtabpop

#Dist of RT in study sample

df_study$RT<-ifelse((df_study$Z5==1 | (df_study$APOE4==1 & df_study$Z6==1 )),"Doomed", ifelse(df_study$Z1==1, "Causal","Immune") )

RTtabsamp<-t(table(df_study$LowEd,df_study$RT)/c(sum(df_study$LowEd==0),sum(df_study$LowEd==1)))

RTtabsamp

#Save results

results_coll$TrueRRpop[i]<-TrueRRpop

results_coll$TrueRDpop[i]<-TrueRDpop

results_coll$CrudeRRpop[i]<-CrudeRRpop

results_coll$CrudeRDpop[i]<-CrudeRDpop

results_coll$CrudeRRsamp[i]<-CrudeRR_Study

results_coll$CrudeRDsamp[i]<-CrudeRD_Study

results_coll$AdjRRsamp[i]<-AdjRR_Study

results_coll$AdjRDsamp[i]<-AdjRD_Study

results_coll$Doomed_E1pop[i]<-RTtabpop["Doomed","1"]

results_coll$Doomed_E0pop[i]<-RTtabpop["Doomed","0"]

results_coll$Immune_E1pop[i]<-RTtabpop["Immune","1"]

results_coll$Immune_E0pop[i]<-RTtabpop["Immune","0"]

results_coll$Doomed_E1samp[i]<-RTtabsamp["Doomed","1"]

results_coll$Doomed_E0samp[i]<-RTtabsamp["Doomed","0"]

results_coll$Immune_E1samp[i]<-RTtabsamp["Immune","1"]

results_coll$Immune_E0samp[i]<-RTtabsamp["Immune","0"]

results_coll$nstudy[i]<-nrow(df_study)

}

#Calculate 95% CI

mean_CIs<-function(data, estimate){

results<-data.frame(Est=estimate)

results$LCI<-quantile(data[,estimate],probs=.025)

results$mean<-mean(data[,estimate])

results$UCI<-quantile(data[,estimate],probs=.975)

return(results)

}

#Auto-generate a results table

for (i in 1:length(colnames(results_coll))){

if (i==1){

res_tab_coll<-mean_CIs(results_coll,colnames(results_coll)[i])

} else{

res_tab_coll<-rbind(res_tab_coll, mean_CIs(results_coll,colnames(results_coll)[i]))}

}

#Print table of results:

#Population average true, crude, and adjusted RRs and RDs across simulations and their 95% CI

#Sample average crude and adjusted RRs and RDs across simulations and their 95% CI

#Average prevalence of response types within exposure groups in population and sample across simulations and their 95% CI (no causal types because simulating under the null)

res_tab_coll

#Drop all objects except results tables

rm(list=grep("res_tab",ls(),value=TRUE,invert=TRUE))

####################################################

##Section 4: Simulating Effect Modification

####################################################

niterations<-1000

blanks<-rep(NA,niterations)

results_EM<-data.frame(

TrueRR=blanks, TrueRD=blanks,

TrueRR_APOE1=blanks, TrueRD_APOE1=blanks,

TrueRR_APOE0=blanks, TrueRD_APOE0=blanks,

CrudeRR=blanks, CrudeRD=blanks,

CrudeRR_APOE1=blanks, CrudeRD_APOE1=blanks,

CrudeRR_APOE0=blanks, CrudeRD_APOE0=blanks,

Doomed_E1=blanks,Causal_E1=blanks, Immune_E1=blanks,

Doomed_E0=blanks,Causal_E0=blanks, Immune_E0=blanks,

Doomed_E1_APOE1=blanks,Causal_E1_APOE1=blanks, Immune_E1_APOE1=blanks,

Doomed_E0_APOE1=blanks,Causal_E0_APOE1=blanks, Immune_E0_APOE1=blanks,

Doomed_E1_APOE0=blanks,Causal_E1_APOE0=blanks, Immune_E1_APOE0=blanks,

Doomed_E0_APOE0=blanks,Causal_E0_APOE0=blanks, Immune_E0_APOE0=blanks)

#Section 4A: Set prevalence of exogenous variables:

pZ1a<-0.04

pZ2<-0.1

pZ1b<-0.1

pAPOE<-0.25 #the effect modifier of explicit interest

pLowEd<-0.7 #the exposure ("E" in the formulas below)

#Section 4B: Simulate cohort of n=10,000 individuals

sampsize<-10000

set.seed(12345) #set a seed so that results can be replicated

for (i in 1:niterations){

#Simulate exogeneous variables

df<-data.frame(ID=seq(1,sampsize),

Z1a=rbinom(sampsize,1,pZ1a),

Z2=rbinom(sampsize,1,pZ2),

Z1b=rbinom(sampsize,1,pZ1b),

LowEd=rbinom(sampsize,1,pLowEd),

APOE=rbinom(sampsize,1,pAPOE),

Dementia=NA)

#Simulate endogenous variables based on SCCs

df$Dementia<-ifelse((df$LowEd==1 & df$Z1a==1) | df$Z2==1 | (df$LowEd==1 & df$APOE==1 & df$Z1b==1) ,1,0) #whether an individual has dementia or not (the outcome, "Y") depends on their value of causal components in the pies whose completion leads to dementia

#Section 4C: Calculate effects

#True effects (in the overall sample and within strata of modifier, APOE)

TrueRR<-(1*pZ1a+pZ2+1*pZ1b*pAPOE-

1*pZ1a*pZ2-1*pZ1a*1*pZ1b*pAPOE-pZ2*1*pZ1b*pAPOE+

1*pZ1a*pZ2*1*pZ1b*pAPOE)/(pZ2)

TrueRD<-(1*pZ1a+pZ2+1*pZ1b*pAPOE-

1*pZ1a*pZ2-1*pZ1a*1*pZ1b*pAPOE-pZ2*1*pZ1b*pAPOE+

1*pZ1a*pZ2*1*pZ1b*pAPOE)-(pZ2)

TrueRR_APOE1<-(1*pZ1a+pZ2+1*pZ1b*1-

1*pZ1a*pZ2-1*pZ1a*1*pZ1b*1-pZ2*1*pZ1b*1+

1*pZ1a*pZ2*1*pZ1b*1)/(pZ2)

TrueRD_APOE1<-(1*pZ1a+pZ2+1*pZ1b*1-

1*pZ1a*pZ2-1*pZ1a*1*pZ1b*1-pZ2*1*pZ1b*1+

1*pZ1a*pZ2*1*pZ1b*1)-(pZ2)

TrueRR_APOE0<-(1*pZ1a+pZ2+1*pZ1b*0-

1*pZ1a*pZ2-1*pZ1a*1*pZ1b*0-pZ2*1*pZ1b*0+

1*pZ1a*pZ2*1*pZ1b*0)/(pZ2)

TrueRD_APOE0<-(1*pZ1a+pZ2+1*pZ1b*0-

1*pZ1a*pZ2-1*pZ1a*1*pZ1b*0-pZ2*1*pZ1b*0+

1*pZ1a*pZ2*1*pZ1b*0)-(pZ2)

#Crude estimates

pY_E0<-sum(df$LowEd==0 & df$Dementia==1)/sum(df$LowEd==0)

pY_E1<-sum(df$LowEd==1 & df$Dementia==1)/sum(df$LowEd==1)

CrudeRR<-pY_E1/pY_E0

CrudeRD<-pY_E1-pY_E0

#Calculate crude estimates in strata of APOE

pY_E0_APOE1<-sum(df$LowEd==0 & df$Dementia==1 & df$APOE==1)/sum(df$LowEd==0 & df$APOE==1)

pY_E1_APOE1<-sum(df$LowEd==1 & df$Dementia==1 & df$APOE==1)/sum(df$LowEd==1 & df$APOE==1)

pY_E0_APOE0<-sum(df$LowEd==0 & df$Dementia==1 & df$APOE==0)/sum(df$LowEd==0 & df$APOE==0)

pY_E1_APOE0<-sum(df$LowEd==1 & df$Dementia==1 & df$APOE==0)/sum(df$LowEd==1 & df$APOE==0)

CrudeRR_APOE1<-pY_E1_APOE1/pY_E0_APOE1

CrudeRD_APOE1<-pY_E1_APOE1-pY_E0_APOE1

CrudeRR_APOE1

CrudeRD_APOE1

CrudeRR_APOE0<-pY_E1_APOE0/pY_E0_APOE0

CrudeRD_APOE0<-pY_E1_APOE0-pY_E0_APOE0

CrudeRR_APOE0

CrudeRD_APOE0

#For effect modification scenario we expect Crude measures = True measures because there are no sources of non-exchangeability

#Section 4D: "Under the hood" (define response types based on SCCs)

#Response types in total study sample

df$RT<-ifelse(df$Z2==1,"Doomed",ifelse(df$Z1a==1 | (df$APOE==1 & df$Z1b==1), "Causal","Immune") )

RTtab<-t(table(df$LowEd,df$RT)/c(sum(df$LowEd==0),sum(df$LowEd==1)))

RTtab

#Response types among those with APOE in the study sample

df_APOE1<-df[df$APOE==1,]

RTtab_APOE1<-t(table(df_APOE1$LowEd,df_APOE1$RT)/c(sum(df_APOE1$LowEd==0),sum(df_APOE1$LowEd==1)))

RTtab_APOE1

#Response types among those without APOE in the study sample

df_APOE0<-df[df$APOE==0,]

RTtab_APOE0<-t(table(df_APOE0$LowEd,df_APOE0$RT)/c(sum(df_APOE0$LowEd==0),sum(df_APOE0$LowEd==1)))

RTtab_APOE0

#Save results

results_EM$TrueRR[i]<-TrueRR

results_EM$TrueRD[i]<-TrueRD

results_EM$TrueRR_APOE1[i]<-TrueRR_APOE1

results_EM$TrueRD_APOE1[i]<-TrueRD_APOE1

results_EM$TrueRR_APOE0[i]<-TrueRR_APOE0

results_EM$TrueRD_APOE0[i]<-TrueRD_APOE0

results_EM$CrudeRR[i]<-CrudeRR

results_EM$CrudeRD[i]<-CrudeRD

results_EM$CrudeRR_APOE1[i]<-CrudeRR_APOE1

results_EM$CrudeRD_APOE1[i]<-CrudeRD_APOE1

results_EM$CrudeRR_APOE0[i]<-CrudeRR_APOE0

results_EM$CrudeRD_APOE0[i]<-CrudeRD_APOE0

results_EM$Doomed_E1[i]<-RTtab["Doomed","1"]

results_EM$Doomed_E0[i]<-RTtab["Doomed","0"]

results_EM$Causal_E1[i]<-RTtab["Causal","1"]

results_EM$Causal_E0[i]<-RTtab["Causal","0"]

results_EM$Immune_E1[i]<-RTtab["Immune","1"]

results_EM$Immune_E0[i]<-RTtab["Immune","0"]

results_EM$Doomed_E1_APOE1[i]<-RTtab_APOE1["Doomed","1"]

results_EM$Doomed_E0_APOE1[i]<-RTtab_APOE1["Doomed","0"]

results_EM$Causal_E1_APOE1[i]<-RTtab_APOE1["Causal","1"]

results_EM$Causal_E0_APOE1[i]<-RTtab_APOE1["Causal","0"]

results_EM$Immune_E1_APOE1[i]<-RTtab_APOE1["Immune","1"]

results_EM$Immune_E0_APOE1[i]<-RTtab_APOE1["Immune","0"]

results_EM$Doomed_E1_APOE0[i]<-RTtab_APOE0["Doomed","1"]

results_EM$Doomed_E0_APOE0[i]<-RTtab_APOE0["Doomed","0"]

results_EM$Causal_E0_APOE0[i]<-RTtab_APOE0["Causal","0"]

results_EM$Causal_E1_APOE0[i]<-RTtab_APOE0["Causal","1"]

results_EM$Immune_E1_APOE0[i]<-RTtab_APOE0["Immune","1"]

results_EM$Immune_E0_APOE0[i]<-RTtab_APOE0["Immune","0"]

}

#Calculate 95% CI

mean_CIs<-function(data, estimate){

results<-data.frame(Est=estimate)

results$LCI<-quantile(data[,estimate],probs=.025)

results$mean<-mean(data[,estimate])

results$UCI<-quantile(data[,estimate],probs=.975)

return(results)

}

#Auto-generate a results table

for (i in 1:length(colnames(results_EM))){

if (i==1){

res_tab_EM<-mean_CIs(results_EM,colnames(results_EM)[i])

} else{

res_tab_EM<-rbind(res_tab_EM, mean_CIs(results_EM,colnames(results_EM)[i]))}

}

res_tab_EM

#Drop all objects except results tables

rm(list=grep("res_tab",ls(),value=TRUE,invert=TRUE))

####################################################

##Section 2b: Simulating confounding, non-null

####################################################

niterations<-1000

blanks<-rep(NA,niterations)

results_confb<-data.frame(TrueRR=blanks, TrueRD=blanks,

CrudeRR=blanks, CrudeRD=blanks,

AdjRR=blanks, AdjRD=blanks,

Doomed_E1=blanks,Causal_E1=blanks, Immune_E1=blanks,

Doomed_E0=blanks,Causal_E0=blanks, Immune_E0=blanks)

#Section 2A: Set prevalence of exogenous variables

pQ1<-0.8

pQ2<-0.05

pZ1<-0.04

pZ3<-0.1

pZ4<-0.05

pLowSES<-0.7

#Section 2B: Simulate cohort of n=10,000 individuals

sampsize<-10000

set.seed(12345) #set a seed so that results can be replicated

for (i in 1:niterations){

#Simulate exogeneous variables

df<-data.frame(ID=seq(1,sampsize),

Q1=rbinom(sampsize,1,pQ1),

Q2=rbinom(sampsize,1,pQ2),

LowSES=rbinom(sampsize,1,pLowSES),

Z1=rbinom(sampsize,1,pZ1),

Z3=rbinom(sampsize,1,pZ3),

Z4=rbinom(sampsize,1,pZ4),

LowEd=NA,

Dementia=NA)

#Simulate endogenous variables based on SCCs

df$LowEd<-ifelse((df$LowSES==1 & df$Q1==1) | df$Q2==1,1,0) #in Scenario 2 (confounding), the exposure (LowEd or "E" in the formulas below) is no longer exogenous; each individual's value for LowEd emerges from the values of the components that cause it

df$Dementia<-ifelse((df$LowEd==1 & df$Z1==1) | df$Z3==1 | (df$LowSES==1 & df$Z4==1),1,0) #whether an individual has dementia or not (the outcome, "Y") depends on their value of causal components in the pies whose completion leads to dementia

#Section 2C: Calculate effects

#Truth (true ATE)

TrueRR<-(pZ1+pZ3+pLowSES*pZ4-pZ1*pZ3-pZ1*pLowSES*pZ4-pZ3*pLowSES*pZ4-pZ1*pZ3*pLowSES*pZ4)/(pZ3+pLowSES*pZ4-pZ3*pLowSES*pZ4)

TrueRD<-(pZ1+pZ3+pLowSES*pZ4-pZ1*pZ3-pZ1*pLowSES*pZ4-pZ3*pLowSES*pZ4-pZ1*pZ3*pLowSES*pZ4)-(pZ3+pLowSES*pZ4-pZ3*pLowSES*pZ4)

#Crude estimates

pY_E0<-sum(df$LowEd==0 & df$Dementia==1)/sum(df$LowEd==0)

pY_E1<-sum(df$LowEd==1 & df$Dementia==1)/sum(df$LowEd==1)

CrudeRR<-pY_E1/pY_E0

CrudeRD<-pY_E1-pY_E0

CrudeRR

CrudeRD

#Standardized (adjusted) estimates

pY_E0_C0<-sum(df$LowEd==0 & df$LowSES==0 & df$Dementia==1)/sum(df$LowEd==0 & df$LowSES==0)

pY_E0_C1<-sum(df$LowEd==0 & df$LowSES==1 & df$Dementia==1)/sum(df$LowEd==0 & df$LowSES==1)

pY_E1_C0<-sum(df$LowEd==1 & df$LowSES==0 & df$Dementia==1)/sum(df$LowEd==1 & df$LowSES==0)

pY_E1_C1<-sum(df$LowEd==1 & df$LowSES==1 & df$Dementia==1)/sum(df$LowEd==1 & df$LowSES==1)

pC1<-nrow(df[df$LowSES==1,])/nrow(df)

pC0<-1-pC1

pY_E1_std<-pY_E1_C1*pC1+pY_E1_C0*pC0

pY_E0_std<-pY_E0_C1*pC1+pY_E0_C0*pC0

AdjRR <- pY_E1_std/pY_E0_std

AdjRD <- pY_E1_std-pY_E0_std

AdjRR

AdjRD

#Section 2D: "Under the hood" (define response types based on SCCs)

df$RT<-ifelse((df$Z3==1 | (df$LowSES==1 & df$Z4==1 )),"Doomed", ifelse(df$Z1==1, "Causal","Immune") )

RTtab<-t(table(df$LowEd,df$RT)/c(sum(df$LowEd==0),sum(df$LowEd==1)))

RTtab

#Store results

results_confb$TrueRR[i]<-TrueRR

results_confb$TrueRD[i]<-TrueRD

results_confb$CrudeRR[i]<-CrudeRR

results_confb$CrudeRD[i]<-CrudeRD

results_confb$AdjRR[i]<-AdjRR

results_confb$AdjRD[i]<-AdjRD

results_confb$Doomed_E1[i]<-RTtab["Doomed","1"]

results_confb$Doomed_E0[i]<-RTtab["Doomed","0"]

results_confb$Causal_E1[i]<-RTtab["Causal","1"]

results_confb$Causal_E0[i]<-RTtab["Causal","0"]

results_confb$Immune_E1[i]<-RTtab["Immune","1"]

results_confb$Immune_E0[i]<-RTtab["Immune","0"]

}

#Calculate 95% CI

mean_CIs<-function(data, estimate){

results<-data.frame(Est=estimate)

results$LCI<-quantile(data[,estimate],probs=.025)

results$mean<-mean(data[,estimate])

results$UCI<-quantile(data[,estimate],probs=.975)

return(results)

}

#Auto-generate a results table

for (i in 1:length(colnames(results_confb))){

if (i==1){

res_tab_confb<-mean_CIs(results_confb,colnames(results_confb)[i])

} else{

res_tab_confb<-rbind(res_tab_confb, mean_CIs(results_confb,colnames(results_confb)[i]))}

}

#Print table of results:

#Average true, crude, and adjusted RRs and RDs across simulations and their 95% CI

#Average prevalence of response types within exposure groups across simulations and their 95% CI (no causal types because simulating under the null)

res_tab_confb

#Drop all objects except results tables

rm(list=grep("res_tab",ls(),value=TRUE,invert=TRUE))
