# Supplementary material for: Sufficient component cause simulations: an underutilized epidemiologic teaching tool
Source: Front Epidemiol. 2023 Nov 10;3:1282809. doi: 10.3389/fepid.2023.1282809 (PMC10906966; doi:10.3389/fepid.2023.1282809)
Supplement: Supplementary file 1 [file Datasheet1.zip › Appendix 2. Confounding.docx]

**Appendix 2. Confounding**

| **DAG** | **SCC model equivalent of DAG** | **Parameter inputs from simulation** | **True**  **effects** |
| --- | --- | --- | --- |
| 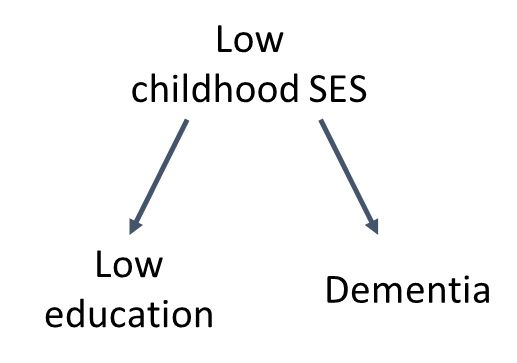 | 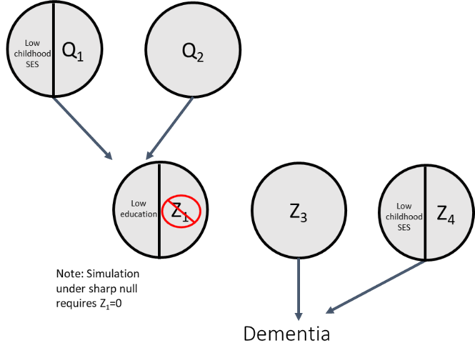 | Prevalence of exogenous variables in simulation:  pQ1=0.8  pQ2=0.2  pZ1=0  pZ3=0.1  pZ4=0.15  pLowSES=0.7 | RR = 1.0  RD = 0.0 |

**I. Estimate crude measures**

We first compute the crude risk ratio and risk difference as follows:

$${Risk ratio}_{crude}= \frac{P (Dementia|Low Education)}{P (Dementia|\bar{Low Education})}$$

${Risk difference}_{crude}=$ $P (Dementia|Low Education)-P (Dementia|\bar{Low Education}$)

The numerator and denominator quantities can be derived from the relevant component causes in Figure 4B and their parameter values specified in the simulation.

$$P \left( Dementia | Low Education \right)= {P((Z}_{1}\cup Z_{3}\cup(LowSES\cap Z_{4}))|Low Education)$$

= ${P(Z}_{1}\left| Low Education \right)+P\left( Z_{3}|Low Education \right)+\left( P\left( LowSES | Low Education \right)*P\left( Z_{4} | Low Education \right) \right)-$ ${(P(Z}_{1}\left| Low Education \right)*P\left( Z_{3}|Low Education \right))-{(P(Z}_{1}\left| Low Education \right)*P\left( LowSES | Low Education \right)* P\left( Z_{4} | Low Education \right))- {(P(Z}_{3}\left| Low Education \right)*P\left( LowSES | Low Education \right)*P\left( Z_{4} | Low Education \right))+ {\left( {P(Z}_{1} | Low Education \right)*P(Z}_{3}\left| Low Education \right)*P\left( LowSES | Low Education \right)*P\left( Z_{4} | Low Education \right))$

= ${P(Z}_{1})+P\left( Z_{3} \right)+\left( P\left( LowSES | Low Education \right)*P\left( Z_{4} \right) \right)-{(P(Z}_{1})*P\left( Z_{3} \right))-$

$${(P(Z}_{1})*P\left( LowSES | Low Education \right)*P\left( Z_{4} \right))-{(P(Z}_{3})*P\left( LowSES | Low Education \right)*{(P(Z}_{4}))+$$

${{(P(Z}_{1})*P(Z}_{3})*P\left( LowSES | Low Education \right)*{(P(Z}_{4}))$,

$$\mathrm{because}Z_{1}, Z_{3}, Z_{4}\perp Low Education$$

$P \left( Dementia | \bar{Low Education} \right)$ = ${P((Z}_{3}\cup(LowSES\cap Z_{4}))|\bar{Low Education})$

= $P\left( Z_{3}|\bar{Low Education} \right)+\left( P\left( LowSES | \bar{Low Education} \right)*P\left( Z_{4} | \bar{Low Education} \right) \right)-$

${(P(Z}_{3}\left| \bar{Low Education} \right)*P\left( LowSES | \bar{Low Education} \right)*P\left( Z_{4} | \bar{Low Education} \right))$

= $P\left( Z_{3} \right)+\left( P\left( LowSES | \bar{Low Education} \right)*P\left( Z_{4} \right) \right)-\left( P\left( Z_{3} \right)*P\left( LowSES | \bar{Low Education} \right)*P\left( Z_{4} \right) \right)$,

$$because Z_{3}, Z_{4}\perp\bar{Low Education}$$

Because confounding creates a dependency between Low SES (confounder) and Low Education (exposure) this must be accounted for when computing the crude risk ratio and risk difference for dementia (in contrast to the causation example in Appendix 1 where exposure was exogenous and calculation of the crude measures followed directly from the simulation parameter inputs with no additional computation required).

We can compute probabilities for the dependency between Low SES and Low Education using Bayes Theorem where quantities in the numerator and denominator are derived from the relevant component causes in Figure 4B:

$$P\left( Low SES|Low Education \right)=\frac{P\left( Low Education | Low SES \right)*P(Low SES)}{P(Low Education)}$$

$$P\left( Low SES|Low Education \right)=\frac{P\left( \bar{Low Education} | Low SES \right)*P(Low SES)}{P(Low Education)}$$

*Where*:

$P\left( Low Education|Low SES \right)=P\left( Q_{1}\cup Q_{2} | Low SES \right)=P(Q_{1}\cup Q_{2})$, because $Q_{1},Q_{2} \perp Low SES$

= 0.8 + 0.2 – 0.8*0.2

= 0.84

$$P\left( \bar{Low Education}|Low SES \right)=1-0.84$$

= 0.16

$$P\left( Low Education \right)=P(\left( Low SES\cap Q_{1})\cup Q_{2} \right)$$

= (0.7*0.8) + (0.2) – (0.7*0.8*0.2)

= 0.648

$$P\left( \bar{Low Education} \right)=1-P(Low Education)$$

= 1 – 0.648

= 0.352

$P\left( Low SES \right)$ = 0.7

*Thus*:

$$P\left( Low SES|Low Education \right)=\frac{P\left( Low Education | Low SES \right)*P(Low SES)}{P(Low Education)}$$

$$=\frac{0.84*0.7}{0.648}$$

= 0.9074

$$P\left( Low SES|Low Education \right)=\frac{P\left( \bar{Low Education} | Low SES \right)*P(Low SES)}{P(Low Education)}$$

$$=\frac{0.16*0.7}{0.352}$$

= 0.3182

We now have all of the necessary probabilities to compute the crude measures in the sample. In the numerator we include all ways in which someone with low education could get dementia (making sure to account for the dependency between low SES and low education in the formula). The denominator includes all ways in which someone without low education could get dementia. Note that because the prevalence of Z_1_ was set to 0, we can eliminate these terms, where relevant, from the equations below.

$$P \left( Dementia | Low Education \right)={P((Z}_{1}\cup Z_{3}\cup(LowSES\cap Z_{4}))|Low Education)$$

$$= P((Z_{1}\cup Z_{3}\cup(LowSES\cap Z_{4}))|Low Education)$$

$$=P\left( Z_{3} \right)+\left( P\left( LowSES | Low Education \right)*P\left( Z_{4} \right) \right)-$$

$${(P(Z}_{3})*P\left( LowSES | Low Education \right)*{(P(Z}_{4}))$$

= 0.10 + 0.9074*0.15 – 0.10*0.9074*0.15

= 0.222499

$P (Dementia|\bar{Low Education}=$ ${P((Z}_{3}\cup(LowSES\cap Z_{4}))|\bar{Low Education})$

= $P\left( Z_{3} \right)+\left( P\left( LowSES | \bar{Low Education} \right)*P\left( Z_{4} \right) \right)-$

$\left( P\left( Z_{3} \right)*P\left( LowSES | \bar{Low Education} \right)*P\left( Z_{4} \right) \right)$

= 0.10 + 0.3182*0.15 – 0.10*0.3182*0.15

= 0.142957

*Thus:*

$${Risk ratio}_{crude}= \frac{P (Dementia|Low Education)}{P (Dementia|\bar{Low Education}}$$

= 0.222499/0.142957

= 1.5564

${Risk differece}_{crude}=P (Dementia|Low Education)- P (Dementia|\bar{Low Education} )$

= 0.222499 – 0.142957

= 0.07954

Note that the 22.2% prevalence of dementia among those with low education and the 14.3% prevalence of dementia among those without low education matches the distribution of the doomed response types within exposure groups in Table 3. This makes sense: because we simulated under the sharp null, the only response types contributing to someone getting the outcome in our sample are the doomed. This imbalance of doomed response types between exposure shows there is non-exchangeability in the sample (due to confounding), which creates a crude risk ratio and risk difference in the sample that do not match the truth (no effect).

**II. Correcting for confounding bias through standardization**

To correct for confounding, we need to standardize by the distribution of the confounder, low SES, which requires the calculation of a few more probabilities. We will use standardization to compute the causal risk ratio. The formula for the standardized risk ratio is as follows:

$${Risk ratio}_{standardized}=$$

$$\frac{P \left( Dementia | Low Education, Low SES \right)P\left( Low SES \right)+ P \left( Dementia | Low Education,\bar{Low SES} \right)P\left( \bar{Low SES} \right)}{P \left( Dementia | \bar{Low Education}, Low SES \right)P\left( Low SES \right)+ P \left( Dementia | \bar{Low Education},\bar{Low SES} \right)P\left( \bar{Low SES} \right)}$$

Again, we can derive the probabilities of interest from the component causes in the figure as follows (and because the prevalence of Z_1_ was set to 0, we can eliminate these terms, where relevant, from the equations below):

$$P \left( Dementia | Low Education, Low SES \right)={P(Z}_{1}\cup Z_{3}\cup Z_{4}\left| Low Education, Low SES \right)$$

$$={P(Z}_{1}\cup Z_{3}\cup Z_{4}), because {Z_{1},Z}_{3}, Z_{4}\perp Low Education$$

$$and {Z_{1},Z}_{3}, Z_{4}\perp Low SES$$

= $P({Z_{1}\cup Z}_{3}\cup Z_{4})$

= *0.10 + 0.15 – 0.10*0.15*

= *0.235*

$P \left( Dementia | Low Education,\bar{Low SES} \right)= P(Z_{1}\cup Z_{3}|Low Education,\bar{Low SES})$

= $P\left( Z_{1}\cup Z_{3} \right)$*,* $because {Z_{1},Z}_{3}\perp Low Education$

$$and {Z_{1},Z}_{3}\perp\bar{Low SES}$$

= $P\left( Z_{1} \cup Z_{3} \right)$

= *0.10*

$$P \left( Dementia | \bar{Low Education}, Low SES \right)={P(Z}_{3}\cup Z_{4}| \bar{Low Education}, Low SES)$$

= $P\left( Z_{3}\cup Z_{4} \right)$*,* $because {Z_{3},Z}_{4}\perp\bar{Low Education}$

$$and {Z_{3},Z}_{4}\perp Low SES$$

= *0.10 + 0.15 – 0.10*0.15*

= *0.235*

$$P \left( Dementia | \bar{Low Education},\bar{Low SES} \right)= P(Z_{3}|\bar{Low Education},\bar{Low SES})$$

= $P(Z_{3})$*,* $because Z_{3}\perp\bar{Low Education} and Z_{3}\perp\bar{Low SES}$

= *0.10*

*Thus:*

$${Risk ratio}_{adj}= \frac{(0.235)(0.70) + (0.10)(0.30)}{(0.235)(0.70) + (0.10)(0.30)}=1.0$$

$${Risk difference}_{adj}= (0.235)(0.70) + \left( 0.10 \right)\left( 0.30 \right)-(0.235)(0.70) + (0.10)(0.30)=0.0$$

After standardization, the RR_adj_ and RD_adj_ are equal to the true value of 1.0 and 0.0, respectively.
