# Supplementary material for: Sufficient component cause simulations: an underutilized epidemiologic teaching tool
Source: Front Epidemiol. 2023 Nov 10;3:1282809. doi: 10.3389/fepid.2023.1282809 (PMC10906966; doi:10.3389/fepid.2023.1282809)
Supplement: Supplementary file 1 [file Datasheet1.zip › Appendix 3. Collider bias.docx]

**Appendix 3. Collider bias**

| **DAG** | **SCC model equivalent of DAG** | **Parameter inputs from simulation** | **True**  **effects** |
| --- | --- | --- | --- |
| 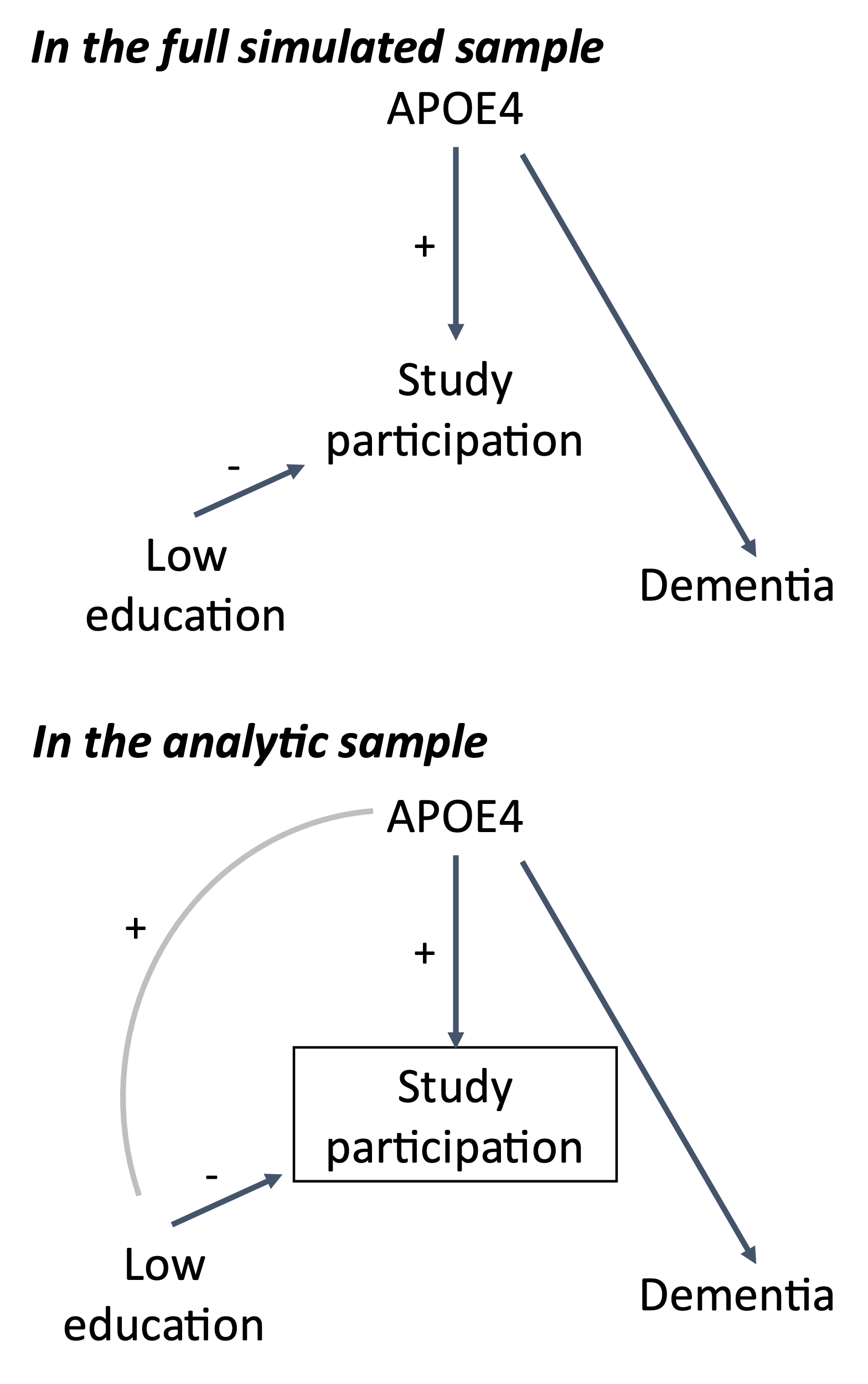 | 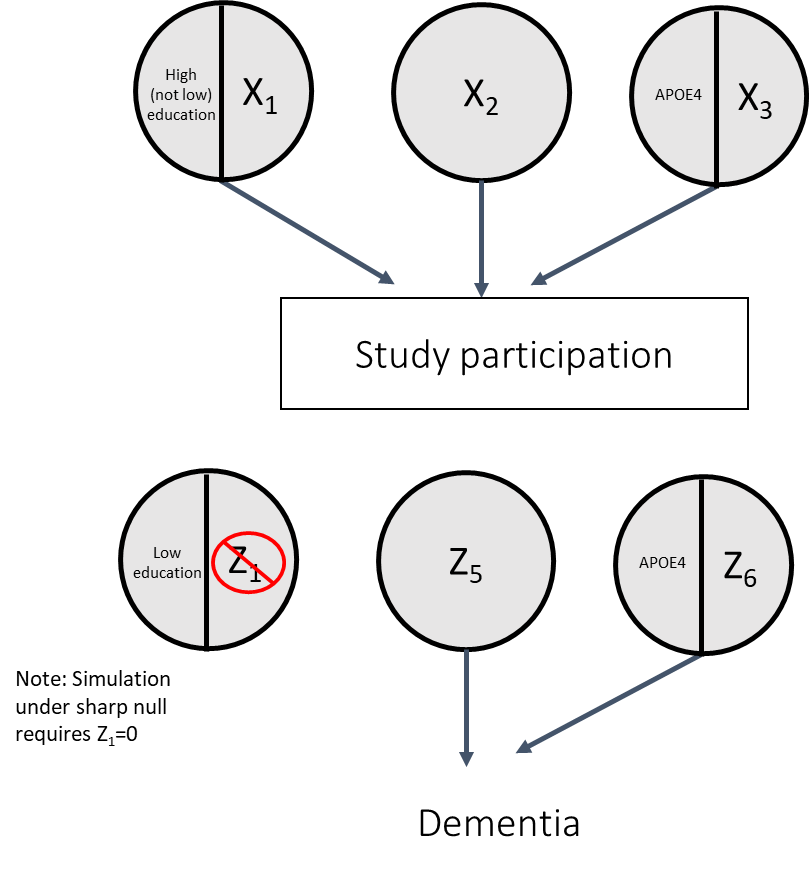 | Prevalence of exogenous variables in simulation:  pX1=0.5  pX2=0.1  pX3=0.5  pZ1=0  pZ5=0.1  pZ6=0.4  pAPOE4=0.25  pLowEd=0.7 | RR = 1.0  RD = 0.0 |

**I. Estimates in the full simulated sample**

We first compute the crude risk ratio and risk difference in the full simulated sample (“full sample”) as follows:

$${Full sample risk ratio}_{crude}= \frac{P (Dementia|Low Education)}{P (Dementia|\bar{Low Education})}$$

${Full sample risk difference}_{crude}=$ $P (Dementia|Low Education)-P (Dementia|\bar{Low Education}$

The numerator and denominator quantities for this formula can be derived from the relevant causal components in Figure 5B and their parameter values specified in the simulation:

`

$P (Dementia|Low Education)$ = P(${(Z}_{1}\cup Z_{5}\cup{(APOE4\cap Z}_{6}))|Low Education)$

= P($Z_{1}\cup Z_{5}\cup{(APOE4\cap Z}_{6}))$, ${because Z}_{1},Z_{5}, APOE4,Z_{6}\perp Low Education$

= $P(Z_{1}\cup Z_{5}\cup{(APOE4\cap Z}_{6}))$ *#P(Z_1_) is eliminated from formula because its prevalence is 0*

= 0.1 + (0.25*0.40) – (0.1*0.25*0.40)

= 0.19

$P (Dementia|\bar{Low Education)}$ = P(($Z_{5}\cup{(APOE4\cap Z}_{6}))|\bar{Low Education})$

= P($Z_{5}\cup{(APOE4\cap Z}_{6}))$, ${because Z}_{5}, APOE4,Z_{6}\perp\bar{Low Education}$

= 0.1 + (0.25*0.40) – (0.1*0.25*0.40)

= 0.19

Thus:

${Full sample risk ratio}_{crude}=\frac{0.19}{0.19}=1.0$

${Full sample risk difference}_{crude}= 0.19-0.19=0.0$

In Table 4 we see that in our simulations, the prevalence of doomed response types in the full simulated sample is also 0.19 among individuals exposed and unexposed to low education. Again, for ease of demonstration, we simulate under the sharp null; thus, the prevalence of the outcome in each exposure group is entirely determined by doomed response types i.e., P(Dementia|Low Education) = 0.19 and analogously the prevalence of doomed risk type in Table 4 is 0.19. Thus, in the full simulated sample there is exchangeability, and the crude RR and RD equal the true values dictated by the data generating mechanism.

**II. Estimates in the analytic sample**

The crude risk ratio and risk difference in the analytic sample (the target population for our estimand) are computed as follows, where S indicates being in the analytic sample and $\bar{\text{S}}$ indicates not:

$${Analytic sample risk ratio}_{crude}= \frac{P (Dementia|Low Education,S)}{P (Dementia|\bar{Low Education},S)}$$

${Analytic sample risk difference}_{crude}= P (Dementia|Low Education, S)$ - $P (Dementia|\bar{Low Education}, S)$

Because High Education (or, equivalently, being *unexposed* to low education) and APOE4 are both causes of study participation, restricting to study participation creates a dependency between Low Education and APOE4 *in the analytic sample*. When we compute crude measures in the analytic sample, we must consider this dependency. These quantities can be derived from the relevant component causes in Figure 5B:

$$P \left( Dementia | Low Education, S \right)= {P((Z}_{1}\cup Z_{5}\cup\left( APOE4 \cap Z_{6} \right))|Low Education, S)$$

= ${P(Z}_{1}\left| Low Education,S \right)+P\left( Z_{5}|Low Education,S \right)+\left( P\left( APOE4 | Low Education,S \right)*P\left( Z_{6} | Low Education,S \right) \right)-$ ${(P(Z}_{1}\left| Low Education,S \right)*P\left( Z_{5}|Low Education,S) \right)-{(P(Z}_{1}\left| Low Education,S \right)*P\left( APOE4 | Low Education,S \right)* P\left( Z_{6} | Low Education,S \right))-{(P(Z}_{5}\left| Low Education,S \right)*P\left( APOE4 | Low Education,S \right)*P\left( Z_{6} | Low Education,S \right))+ {\left( {P(Z}_{1} | Low Education,S \right)*P(Z}_{5}\left| Low Education,S \right)*P\left( APOE4 | Low Education,S \right)*P\left( Z_{6} | Low Education,S \right))$

= ${P(Z}_{1})+P\left( Z_{5} \right)+\left( P\left( APOE4 | Low Education,S \right)*P\left( Z_{6} \right) \right)-{(P(Z}_{1})*P\left( Z_{5} \right))-$

$${(P(Z}_{1})*P\left( APOE4 | Low Education,S \right)*P\left( Z_{6} \right))-{(P(Z}_{5})*P\left( APOE4 | Low Education,S \right)*{(P(Z}_{6}))+$$

${{(P(Z}_{1})*P(Z}_{5})*P\left( APOE4 | Low Education,S \right)*{(P(Z}_{6}))$, $because Z_{1}, Z_{5}, Z_{6}\perp Low Education$

$$and Z_{1}, Z_{5}, Z_{6}\perp S$$

$$P \left( Dementia | \bar{Low Education}, S \right)= {P((Z}_{5}\cup\left( APOE4\cap Z_{6} \right))|\bar{Low Education}, S)$$

=$P\left( Z_{5}|\bar{Low Education},S \right)+\left( P\left( APOE4 | \bar{Low Education},S \right)*P\left( Z_{6} | \bar{Low Education},S \right) \right)-$

$${(P(Z}_{5}\left| \bar{Low Education},S \right)*\left( P\left( APOE4 | \bar{Low Education},S \right)*P\left( Z_{6} | \bar{Low Education},S \right) \right)$$

= $P\left( Z_{5} \right)+\left( P\left( APOE4 | \bar{Low Education},S \right)*P\left( Z_{6} \right) \right)-{(P(Z}_{5})*P\left( APOE4 | \bar{Low Education},S \right)*{(P(Z}_{6}))$,

$$because Z_{5}, Z_{6}\perp\bar{Low Education}$$

$$and Z_{5}, Z_{6}\perp S$$

Thus, we must also compute the following probabilities:

$P\left( APOE4 | Low Education, S \right)$ *i.e., the probability you have APOE4 given you are in the study sample and have low education*

$P\left( APOE4 \right|\bar{Low Education}, S)$ *i.e., the probability you have APOE4 given you are in the study sample and have high education*

We can compute these probabilities using Bayes Theorem where quantities in the numerator and denominator are derived from derived from the relevant component causes in Figure 5B:

$$P\left( APOE4 | Low Education, S \right)=\frac{P\left( S | APOE4, Low Education \right)P\left( Low Education | APOE4 \right)P(APOE4)}{P\left( S | Low Education \right)P(Low Education)}$$

*Where*:

$P\left( S | APOE4, Low Education \right)={P(X}_{2}\cup X_{3}|APOE4,Low Education)$

$={P(X}_{2}\cup X_{3}), because X_{2}, X_{3}\perp APOE4$

$and X_{2}, X_{3}\perp Low Education$

= *0.1 + 0.5 – 0.1*0.5*

= *0.55*

$P\left( S | Low Education \right)$ = ${P((X}_{2}\cup{(APOE4\cap X}_{3}))|Low Education)$

$={P(X}_{2}\cup{(APOE4\cap X}_{3})), because X_{2}, {APOE4,X}_{3}\perp Low Education$

*#Because low/high education and APOE4 are independent in the full simulated sample (i.e., before selection)*

= *0.1 + (0.25*0.5) – 0.1*0.25*0.5*

= *0.2125*

$P\left( Low Education | APOE4 \right)=P\left( Low Education \right)$*,* $because Low Education\perp APOE4$

$$=0.70$$

*Thus*:

$$P\left( APOE4 | Low Education, S \right)=\frac{\left( 0.55 \right) x \left( 0.70 \right) x (0.25)}{\left( 0.2125 \right) x \left( 0.70 \right)}= \frac{\left( 0.55 \right) x (0.25)}{\left( 0.2125 \right)}=0.6471$$

Intuitively, this probability of APOE4 within the analytic sample makes sense. Having high education or APOE4 are two possible ways that an individual makes it into the analytic sample. If you are in the analytic sample and you do *not* have high education, this should mean that, within the analytic sample, your probability of having APOE4 is higher than 0.25.

We can likewise perform these calculations for those with high education (i.e., not low education):

$$P\left( APOE4 | \bar{Low Education}, S \right)=\frac{P\left( S | APOE4, \bar{Low Education} \right)P\left( \bar{Low Education} | APOE4 \right)P(APOE4)}{P\left( S | \bar{Low Education} \right)P(\bar{Low Education})}$$

*Where*:

$P\left( S | APOE4,\bar{Low Education} \right)$ = $P(X_{1}\cup X_{2}\cup X_{3}|APOE4,\bar{Low Education}$)

$=P\left( X_{1}\cup X_{2}\cup X_{3} \right), because X_{1}, X_{2}, X_{3}\perp APOE4$

$and X_{1}, X_{2}, X_{3}\perp\bar{Low Education}$

*= 0.5 + 0.1 + 0.5 – (0.1*0.5) – (0.1*0.5) – (0.5*0.5) + (0.5*0.5*0.1)*

*= 0.775*

$P\left( S | \bar{Low Education} \right)$ = ${{P((X}_{1}\cup X}_{2}\cup{(APOE4\cap X}_{3}))|\bar{Low Education})$

$={{P(X}_{1}\cup X}_{2}\cup{(APOE4\cap X}_{3})), because X_{1},X_{2}, {APOE4,X}_{3}\perp\bar{Low Education}$

*#Because low/high education and APOE4 are independent in the full simulated sample (i.e., before selection)*

= *0.5+0.1+(0.25*0.5)–(0.5*0.1)–(0.1*0.125)–(0.5*0.125)+(0.5*0.1*0.125)*

= *0.60625*

$P\left( \bar{Low Education} | APOE4 \right)=P\left( \bar{Low Education} \right), because \bar{Low Education}\perp APOE4$

$$=0.30$$

*Therefore*:

$$\frac{\left( 0.775 \right) x \left( 0.30 \right) x (0.25)}{\left( 0.60625 \right) x \left( 0.30 \right)}= \frac{\left( 0.775 \right) x (0.25)}{\left( 0.60625 \right)}=0.31959$$

We now have what we need to compute the risk ratio in the analytic sample:

$$P \left( Dementia | Low Education, S \right)= {P((Z}_{1}\cup Z_{5}\cup(APOE4\cap Z_{6}))|Low Education,S)$$

$$= P((Z_{1}\cup Z_{5}\cup(APOE4\cap Z_{6}))|Low Education,S)$$

$$=P\left( Z_{5} \right)+\left( P\left( APOE4 | Low Education,S \right)*P\left( Z_{6} \right) \right)-$$

$${(P(Z}_{5})*P\left( APOE4 | Low Education,S \right)*{(P(Z}_{6}))$$

$= 0.10+(0.6471*0.40)$ – (0.10 * 0.6471 * 0.4)

**= 0.33296**

$$P \left( Dementia | \bar{Low Education}, S \right)= {P((Z}_{5}\cup(APOE4\cap Z_{6}))|\bar{Low Education},S)$$

= $P\left( Z_{5} \right)+\left( P\left( APOE4 | \bar{Low Education},S \right)*P\left( Z_{6} \right) \right)-$

$\left( P\left( Z_{5} \right)*P\left( APOE4 | \bar{Low Education},S \right)*P\left( Z_{6} \right) \right)$

$= 0.10+(0.31959*0.40)$ – (0.10 * $0.31959$ * 0.4)

**= 0.2151**

$${Sample risk ratio}_{crude}= \frac{P (Dementia|Low Education, S)}{P (Dementia|\bar{Low Education}, S)}= \frac{0.333}{0.215}= \boldsymbol{1.55}$$

${Sample risk difference}_{crude}= P (Dementia|Low Education, S)$ - $P (Dementia|\bar{Low Education}, S)$

= 0.333 – 0.215 = 0.118

**= 0.12**

Again, because we simulate under the sharp null, these outcome probabilities are entirely determined by the doomed response types. We see in Table 4 that, indeed, within the analytic sample, the prevalence of dementia (i.e., of doomed response types) among those with low education is 33.3% and among those with high education (i.e., without low education) is 21.6%. Thus, there is non-exchangeability due to collider bias within the analytic sample, and this bias is reflected in the crude estimates.

**III. Correcting for collider bias in study sample estimates through standardization**

In this example, if we knew both high education and APOE4 were the causes of study participation (the collider), we could compute a risk ratio for the effect of education on dementia that corrected for collider bias by standardizing to the distribution of the collider-inducing variable, APOE4, in the analytic sample and obtain the analytic sample adjusted (i.e., standardized) risk ratio and risk difference as follows:

${Analytic sample risk ratio}_{standardized}$*=*

$$\frac{P \left( Dementia | Low Education, APOE4,S \right)P\left( APOE4|S \right)+ P \left( Dementia | Low Education,\bar{APOE4},S \right)P\left( \bar{APOE4}|S \right)}{P \left( Dementia | \bar{Low Education},APOE4,S \right)P\left( APOE4|S \right)+ P \left( Dementia | \bar{Low Education},\bar{APOE4},S \right)P\left( \bar{APOE4}|S \right)}$$

${Analytic sample risk difference}_{standardized}$*=*

$$P \left( Dementia | Low Education, APOE4,S \right)P\left( APOE4,S \right)+ P \left( Dementia | Low Education,\bar{APOE4},S \right)P\left( \bar{APOE4},S \right)$$

$$-P \left( Dementia | \bar{Low Education},APOE4,S \right)P\left( APOE4,S \right)+ P \left( Dementia | \bar{Low Education},\bar{APOE4},S \right)P\left( \bar{APOE4},S \right)$$

Again, we can derive the probabilities of interest from the component causes in the figure as follows (and because the prevalence of Z_1_ was set to 0, we can eliminate these terms, where relevant, from the equations below):

*Where*:

$$P \left( Dementia | Low Education, APOE4,S \right)={P(Z}_{1}\cup Z_{5}\cup Z_{6}|Low Education, APOE4,S)$$

$$={P(Z}_{1}\cup Z_{5}\cup Z_{6}), becauseZ_{1}, Z_{5}, Z_{6}\perp Low Education$$

$${and Z}_{1}, Z_{5}, Z_{6}\perp APOE4$$

$${and Z}_{1}, Z_{5}, Z_{6}\perp S$$

= ${P(Z_{1}\cup Z}_{5}\cup Z_{6})$

= *0.10 + 0.40 – 0.10*0.40*

= *0.46*

$$P \left( Dementia | Low Education,\bar{APOE4},S \right)={P(Z}_{1}\cup Z_{5}|Low Education, \bar{APOE4},S)$$

$$={P(Z}_{1}\cup Z_{5}), becauseZ_{1}, Z_{5}\perp Low Education$$

$${and Z}_{1}, Z_{5}\perp\bar{APOE4}$$

$${and Z}_{1}, Z_{5}\perp S$$

= $P(Z_{1}\cup Z_{5})$

= *0.10*

$$P \left( Dementia | \bar{Low Education}, APOE4,S \right)={P(Z}_{5}\cup Z_{6}|\bar{Low Education}, APOE4, S)$$

$$={P(Z}_{5}\cup Z_{6}), because Z_{5}, Z_{6}\perp\bar{Low Education}$$

$${and Z}_{5}, Z_{6}\perp APOE4$$

$${and Z}_{5}, Z_{6}\perp S$$

= *0.10 + 0.40 – 0.10*0.40*

= *0.46*

$$P \left( Dementia | \bar{Low Education},\bar{APOE4},S \right)= {P(Z}_{5}\left| \bar{Low Education},\bar{APOE4},S \right)$$

$$={P(Z}_{5}), because Z_{5}\perp\bar{Low Education}$$

$${and Z}_{5}\perp APOE4$$

$${and Z}_{5}\perp S$$

= *0.10*

*And:*

$$P \left( APOE4 | S \right)= \frac{P\left( S | APOE4 \right)P(APOE4)}{P(S)}$$

*Where:*

$P\left( S | APOE4 \right)=P\left( (\left( {\bar{Low Education}\cap X}_{1} \right)\cup X_{2}\cup X_{3})|APOE4) \right)$

$= P\left( \left( {\bar{Low Education}\cap X}_{1} \right)\cup X_{2}\cup X_{3} \right), because \bar{Low Education}$*,*$X_{1},X_{2} {,X}_{3}\perp APOE4$

*#Because low/high education and APOE4 are independent in the full simulated sample (i.e., before selection)*

= (0.3*0.5) + 0.1 + 0.5 – (0.3*0.5*0.1) – (0.1*0.5) – (0.3*0.5*0.5) + (0.3*0.5*0.1*0.5)

= 0.6175

$P(S)$ *=* ${P((\bar{Low Education}\cap X}_{1})\cup X_{2}\cup({APOE4\cap X}_{3}))$

= (0.3*0.5)+0.1+(0.25*0.5)–(0.3*0.5*0.1)–(0.1*0.25*0.5)–(0.3*0.5*0.25*0.5)+(0.3*0.5*0.1*0.25*0.5)

= $0.330625$

$P\left( APOE4 \right)=$0.25

*Thus:*

$$P(APOE4|S)= \frac{0.6175*0.25}{0.330625}=0.4669$$

*and* $P\left( \bar{APOE4} | S \right)= 1-0.4669=0.5331$

We have now computed all quantities required to calculate the standardized risk ratio and risk difference in the analytic sample:

$$Analytic {sample risk ratio}_{standardized}= \frac{\left( 0.46 \right)\left( 0.4669 \right) + \left( 0.10 \right)\left( 0.5331 \right)}{\left( 0.46 \right)\left( 0.4669 \right) + \left( 0.10 \right)\left( 0.5331 \right)}=1.0$$

$${Analytic sample risk difference}_{standardized}= (0.46)(0.4669) + \left( 0.10 \right)\left( 0.5331 \right)-(0.46)(0.4669) + (0.10)(0.5331)=0.0$$

After standardization, the RR and RD in the analytic sample now equal the RR and RD in the full simulated sample and, in both, the RR and RD are equal to the true value of 1.0 and 0.0, respectively.
